# Seasonal Variation in Sea Turtle Density and Abundance in the Southeast Florida Current and Surrounding Waters

**DOI:** 10.1371/journal.pone.0145980

**Published:** 2015-12-30

**Authors:** Caitlin M. Bovery, Jeanette Wyneken

**Affiliations:** Department of Biological Sciences, Florida Atlantic University, Boca Raton, Florida, United States of America; The Evergreen State College, UNITED STATES

## Abstract

Assessment and management of sea turtle populations is often limited by a lack of available data pertaining to at-sea distributions at appropriate spatial and temporal resolutions. Assessing the spatial and temporal distributions of marine turtles in an open system poses both observational and analytical challenges due to the turtles’ highly migratory nature. Surface counts of marine turtles in waters along the southern part of Florida’s east coast were made in and adjacent to the southeast portion of the Florida Current using standard aerial surveys during 2011 and 2012 to assess their seasonal presence. This area is of particular concern for sea turtles as interest increases in offshore energy developments, specifically harnessing the power of the Florida Current. While it is understood that marine turtles use these waters, here we evaluate seasonal variation in sea turtle abundance and density over two years. Density of sea turtles observed within the study area ranged from 0.003 turtles km^-2^ in the winter of 2011 to 0.064 turtles km^-2^ in the spring of 2012. This assessment of marine turtles in the waters off southeast Florida quantifies their in-water abundance across seasons in this area to establish baselines and inform future management strategies of these protected species.

## Introduction

Understanding spatial and temporal distributions is crucial for effective management and conservation strategies of threatened and endangered species. Obtaining such information is especially challenging for highly mobile wildlife that occupy multiple habitats throughout their life cycle. Sea turtles are large marine reptiles that undergo several habitat shifts that are ontogenetic [1–5] and migratory [6–11]. Such habitat changes complicate studies of local and global distributions, necessitating studies that examine in-water distributions at varied spatial and temporal scales [12, 13]. Due to the complex life history of marine turtles [1, 2, 10, 14, 15], knowledge of their spatial and temporal distributions is essential for understanding population structures and focusing conservation efforts. The southeast Atlantic coast of Florida hosts some of the world’s largest sea turtle rookeries [16–18] and provides valuable in-water habitat for various life stages and species [19]. Five of the world’s sea turtle species, including loggerhead (*Caretta caretta*), leatherback (*Dermochelys coriacea*), green (*Chelonia mydas*), Kemp’s ridley (*Lepidochelys kempii*), and hawksbill (*Eretmochelys imbricata*) turtles, use the waters along the southeastern coast of Florida [19]. Each occupies various pelagic and benthic niches within these waters throughout different life stages [1, 20–23].

Of the five species, the loggerhead [13, 24, 25], leatherback [12, 17], and green [18, 26] turtles migrate and nest regularly in increasing numbers [27] along the southeast coast of Florida, while few hawksbill [28, 29] and Kemp’s ridley [28, 30] nests occur annually. Florida’s east coast is important as transitional habitat for hatchlings dispersing offshore [31], as well as developmental habitat for turtles originating from a wide range of natal beaches [23]. Large juvenile and adult loggerhead turtles are captured or observed along Florida’s Atlantic coast year-round [2, 13, 32, 33]. Similarly, juvenile green turtles are consistently present in Florida’s Atlantic waters in a variety of developmental and foraging habitats including nearshore reefs, coastal lagoons, and sea grass beds [33–35]. Most Kemp’s ridley turtles present in Florida’s Atlantic waters are juveniles undergoing coastal foraging migrations [36–39]. Juvenile hawksbills occur along hard-bottom reefs in southeast Florida [29, 40], yet few studies exist assessing their presence year-round. Very little is known about juvenile leatherback turtles once they leave the nesting beaches [12].

Oceanic currents are important physical features of some habitats used by sea turtles [21, 31, 41–43]. Sea turtles often associate with currents during long-distance movements and as a primary foraging area [21, 41, 43, 44], which in turn can influence the distribution of nests along natal beaches [45]. The Florida Current, the southern portion of the Gulf Stream, is a unique oceanic feature that runs swiftly northward between the southeastern Florida coast and the Bahamas at speeds of up to 2 m s^-1^ at its core located approximately 20 km offshore [46]. Due to its proximity to nesting beaches and developmental habitats in south Florida, it is likely that sea turtle migrations intersect the Florida current and its surrounding waters. The potential for alternative energy development on the outer continental shelf and within the Florida current raises questions about the potential impacts on endangered sea turtles and other marine fauna [46]. Fundamental in the assessment of such concerns is a sound understanding of sea turtle distributions and abundances in and around the Florida current. That data gap necessitates fine-scale examination of sea turtle distributions to understand the potential for interactions with alternative energy industries.

Aerial surveys are commonly used to identify distributions of sea turtles over various spatial and temporal scales [47]. Loggerhead, leatherback, green, and Kemp’s ridley turtles have been monitored using aerial surveys in a variety of habitats to assess spatial and temporal variations in presence [48–57] or to estimate density and abundance of a species in a given area [49, 50, 53, 54, 57, 58]. Here we estimate sea turtle density and abundance in a discrete portion of the Florida Current and adjacent waters using systematic aerial survey data and identify trends in the spatial presence and temporal abundance of sea turtles.

## Material and Methods

The Florida Atlantic University IACUC granted a waiver of ethical approval because the animals were not handled, captured or manipulated. The work was authorized under US NMFS Permit Number 14586 “Assessment of sea turtles and marine mammals within the Florida and Gulf Stream Currents in the northern Florida Straits."

### Study area

**Fig 1:**
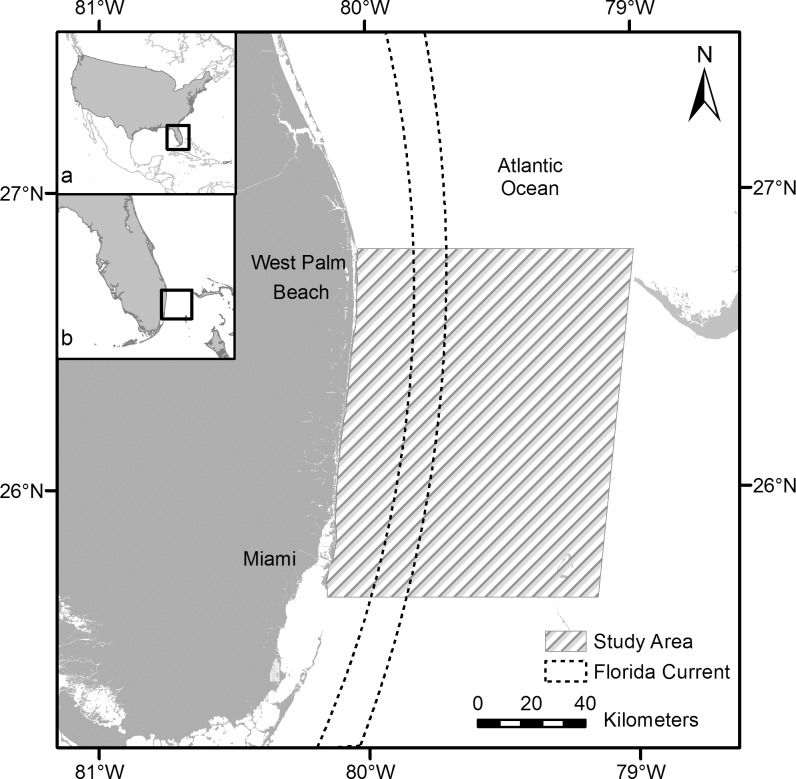
Study area for aerial surveys. Map includes an (a) inset of the United States of America and (b) the state of Florida showing the region for this study. Hatched area indicates extent of the survey area over which aerial surveys were conducted. Dotted lines representing the core of the Florida Current are meant as approximations for reference. Source: ArcWorld Supplement, Florida Fish and Wildlife Conservation Commission-Fish and Wildlife Research Institute.

The study area was defined as waters off the eastern coast of Florida extending south of 26°43′N, (near West Palm Beach, Florida) to north of 25°40′N, near Miami, Florida (Fig 1). This area encompasses the southern portion of the Gulf Stream that runs northward between the western Bahamas and the eastern coast of Florida (hereafter the Florida Current). Our study also includes an area that has been identified as a potential site for oceanic energy interests. Structures in the water column have potential to impact wildlife (at various life stages) that use the Florida Current, as well as coastal habitats westward of the current.

### Aerial surveys

To investigate seasonal presence of marine turtles in this study area, systematic aerial surveys were conducted at approximately monthly intervals from 2011–2012 to complete a total of 22,433 km of survey effort. Surveys in 2011 consisted of 12 parallel transects ranging in length from 40–100 km spaced 8 km apart. In 2012, the surveys were optimized using distance sampling methodology [59] and the survey was redesigned to include 16 parallel transects ranging in length from 35–100 km, spaced 4 km apart. All surveys were flown in a high-wing aircraft (Cessna 337) at a constant altitude of 150 m and groundspeed of 185 km h^-1^. Following line transect theory [59, 60], parallel transects were flown east to west to follow an assumed gradient of sea turtle distributions, and were spaced far enough apart to minimize the likelihood of encountering an individual sea turtle more than once in a single survey.

The crew consisted of a pilot, co-pilot, and two trained observers positioned on opposite sides of the aircraft on each flight. Prior to surveys, observers were trained in survey techniques and their ability to accurately identify species was verified. At the beginning of each transect, environmental conditions were recorded including sea state, glare, and cloud cover to be tested as potential covariates in density estimation. Surveys were only conducted in Beaufort Sea States ≤ 3 to maximize animal visibility. Upon sighting an animal, the position, species, relative size (S, M, L), number of individuals, sighting angle, and sea state were recorded. Position was recorded using a Garmin 12XL GPS unit with external antenna (15 m accuracy). Sighting angle (declination angle) was recorded using hand-held Suunto™ clinometers (PM-5/360PC). When possible, relative size classes of turtles were estimated by the observers and categorized as small, medium, or large relative for each species to provide rough estimates of the life stages present (small juvenile, large juvenile, adult respectively) in these waters. Where size class was ambiguous between small and medium, the turtles were classified as small; similarly, turtles that were between medium and large were classified as large. Size classification was based on observer knowledge and training prior to flights.

Visualizations of the spatial distributions of all sightings of sea turtles from the surveys and the most commonly sighted species, Loggerhead (*Caretta caretta*) and green turtle (*Chelonia mydas*) were constructed to illustrate spatial patterns and assess proximity to shoreline and the Florida Current in our study. One sea turtle sighting was not used due to a poor GPS reading. Dotted lines denoting the Florida Current are approximate locations for reference and were not used in analyses. Seasons are defined as winter (December-February), spring (March-May), summer (June-August), and fall (September-November) to examine trends in presence of sea turtles. All maps were created using ArcGIS® software by ESRI®.

### Density estimation

Density and abundance of sea turtles was estimated using line transect distance analyses. Distance sampling relies on three key underlying assumptions that are important for reliable estimates of density [59, 60]. First, all animals on the transect line are observed. Due to flat windows, the transect line was not visible in our study. To accommodate for violation of this assumption, left-truncation was employed subtracting 82 m from all recorded perpendicular distances to account for the blind area under the plane [50]. This correction effectively shifted the transect line to the observers’ visible range.

Secondly, distance sampling assumes that animals are detected before any movement. In this survey, turtle behavior was recorded so any potential avoidance behavior could be identified to meet the assumption that animals are detected prior to movement. Avoidance behavior was defined conservatively as diving when the plane passed by. Less than 5% of all observed sea turtles were diving.

Lastly, distances are assumed to be measured accurately. To minimize measurement bias, all distances were calculated from the declination angle of the sighting relative to the horizon. This measurement was then used to calculate the perpendicular distance of the animal from the transect line using the formula:

PerpedicularDistance=h*tan(90−α)

where *h* is the altitude of the plane (150 m) and α is the declination angle.

Conventional distance sampling (CDS) and multiple covariate distance sampling (MCDS) analyses were performed using Distance v.6.2 [60]. In CDS methodology, the probability of detection, (detection function), is modeled using the perpendicular distance from the transect line, while MCDS analysis allows the inclusion of covariates in the model of the detection function [59,60]. Density ($\hat{D}$) is then estimated using a Horowitz-Thompson-like estimator [53,61] in the form of:

$$\hat{D}\;=\;\frac{n}{2wL{\hat{P}}_{a}}$$

in CDS analyses and:

$$\hat{D}\;=\;\frac{1}{2wL}\sum_{i=1}^{n}\;\frac{1}{{\hat{P}}_{a}(z_{i})}$$

in MCDS analyses where *w* is half the effective strip width, *L* is the total length of transects, *n* is the number of turtles observed, ${\hat{P}}_{a}$ is the probability of detection for the *i*th turtle within the covered area (*2wL*) given the observed covariates (*z*_*i*_) where applicable. Seasonal estimates of abundance were obtained from the global model of the probability of detection using stratification by season within year.

Uniform, half-normal, and hazard-rate models of the detection function with cosine, simple polynomial, or hermite polynomial adjustments were tested using CDS to determine the best-fit model. Observer, glare, cloud cover, and sea state were considered as covariates using MCDS analysis to test the same models of the detection function for any influence from those factors. Observers were grouped as primary and secondary for all MCDS analyses. Minimum Akaike’s Information Criterion (AIC) was used to select among the candidate CDS and MCDS models.

Key functions tested are uniform (Uni), half-normal (HN), and hazard-rate (HR) with cosine (COS), simple polynomial (SP) or hermite polynomial (HP) adjustment terms. Covariates included in MCDS are observer (OBS), sea state (SS), cloud cover (CC), and glare (GL).

## Results

### Observations

**Fig 2:**
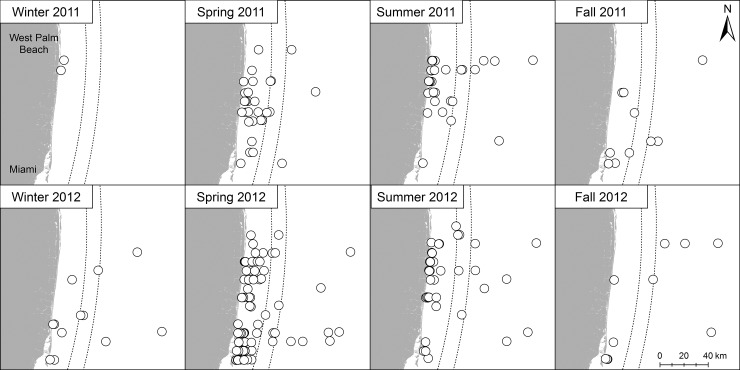
Seasonal sightings of sea turtles from aerial surveys conducted in 2011–2012. Each symbol represents a single sea turtle. Dotted lines represent the approximate location of the core of the Florida Current. Source: Florida Fish and Wildlife Conservation Commission-Fish and Wildlife Research Institute.

**Fig 3:**
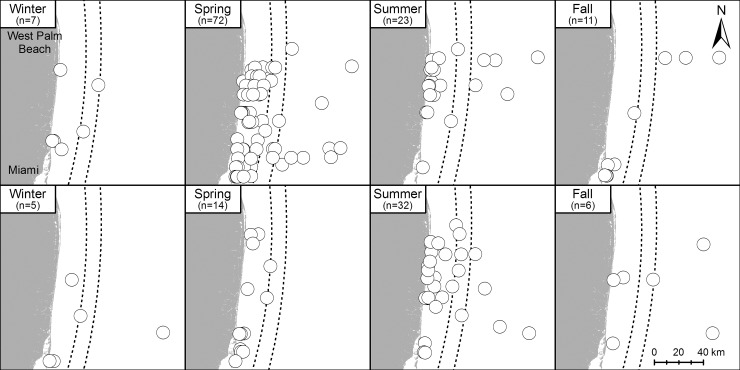
Sightings of the two most commonly observed sea turtle species in 2011–2012 by season. Loggerheads (top panels) and green turtles (bottom panels) sighted in each season during 2011–2012 aerial surveys. Note that the highest numbers of loggerheads sighted is in the spring while the highest numbers of green turtles sighted is in the summer. Each symbol represents a single sea turtle. Dotted lines represent the approximate location of the core of the Florida Current. Source: Florida Fish and Wildlife Conservation Commission-Fish and Wildlife Research Institute.

During this study, 218 sea turtles were observed on effort (2011: n = 79; 2012: n = 139) at a sighting rate of 0.01 turtles observed per km surveyed (Table 1). Four species (80% of all observations) were sighted and identified to species: loggerheads (*Caretta caretta*), green turtles (*Chelonia mydas*), leatherbacks (*Dermochelys coriacea*), and Kemp’s ridleys (*Lepidochelys kempii*). Spatial distributions of sea turtle sightings revealed that 72.8% of observations (n = 158) occurred within 20 km of the shoreline, west of the estimated core of the Florida Current (Fig 2). The most frequently observed species in our study was the loggerhead turtle (n = 113; 52% of all observations) followed by the green turtle (n = 57, 26%). Distributions for both species were similar within our study area (Fig 3). Leatherbacks (1.8%, n = 4) and Kemp’s ridleys (<1%, n = 1) were rare. The low flight altitude allowed for confident identification of most turtles to species. The remaining 20% of sea turtle sightings (n = 43) could not be identified to species due to water clarity and distance from plane, but could be classified as cheloniids (hard-shelled species) and not as leatherbacks. Size classes recorded during the surveys indicated that small juvenile (n = 39), large juvenile (n = 116), and adult (n = 45) life stages were present in these waters.

**Table 1:** Summary of combined aerial survey effort (km), the number of sea turtles sighted (*n*), seasonal estimates of sea turtle density (turtles/km^2^) and abundance with 95% confidence intervals for each estimate including lower and upper confidence limits (LCL and UCL, respectively). Seasons are defined as winter (December-February), spring (March-May), summer (June-August), and fall (September-November).

Year	season	effort (km)	*n*	density ($\hat{D}$)	abundance ($\hat{N}$)	% CV	95% CI ($\hat{D}$)		95% CI ($\hat{N}$)	
							LCL	UCL	LCL	UCL
2011	winter	2481.1	3	0.003	41	74%	0.001	0.012	11	157
	spring	2598.2	30	0.030	392	30%	0.017	0.054	218	705
	summer	2608.4	30	0.030	391	25%	0.018	0.050	237	645
	fall	2612.5	10	0.010	130	40%	0.005	0.022	59	285
2012	winter	3070.5	12	0.010	133	32%	0.006	0.019	72	246
	spring	2957.8	73	0.064	838	18%	0.045	0.092	587	1195
	summer	3036.3	37	0.032	414	19%	0.022	0.047	281	608
	fall	3068.2	12	0.010	133	48%	0.004	0.026	53	333
Total		22,433	207	0.024	313	12%	0.019	0.030	250	393

### Density estimation

**Fig 4:**
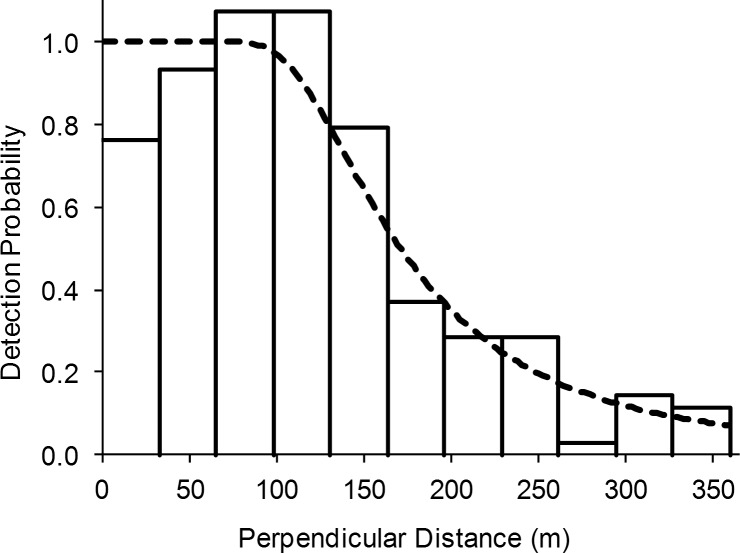
Plot of the detection function for sea turtles based on the AIC selected Conventional Distance Sampling (CDS) model. Histogram represents the probability of detection for each distance interval. The curved line is the detection function, showing the probability that a turtle is observed as a function of distance from the transect line.

After excluding sightings with no declination angle recorded and left-truncating data according to distance sampling methodology, 207 observations were included in the density estimation analyses. Due to low numbers of turtle observations in some seasons and low numbers of some species, observations were pooled across seasons and species to estimate the probability of detection. Despite rigorous MCDS analysis, results indicated that the best model did not include any of the proposed covariates. Model selection using Akaike’s Information Criterion (AIC) identified a hazard-rate model with no adjustments as the best fit for sea turtle detection in this study (Table 2; Fig 4).

**Table 2:** Akaike’s Information Criterion (AIC) values for the conventional distance sampling and multiple covariate distance sampling models of the detection function tested.

	model	adjustment terms	parameters	AIC	ΔAIC
*conventional distance sampling (CDS)*					
	HR	HP*	2	2329.61	0.00
	HR	SP*	2	2329.61	0.00
	HN	SP*	1	2333.73	4.12
	HN	COS*	1	2333.73	4.12
	Uni	COS*	1	2334.04	4.43
	model		parameters	AIC	ΔAIC
*multiple covariate distance sampling (MCDS)*					
	GL		3	2331.64	2.03
	OBS		3	2331.64	2.03
	SS		4	2333.63	4.02
	OBS SS		4	2333.64	4.02
	OBS GL		4	2333.64	4.03
	CC		5	2335.42	5.81
	OBS SS GL		5	2335.62	6.00
	SS GL		5	2335.63	6.02
	OBS SS CC		7	2336.38	6.77
	CC GL		6	2337.42	7.81
	OBS CC		6	2337.43	7.82
	OBS CC GL SS		8	2338.20	8.59
	SS CC		7	2339.35	9.74
	OBS CC GL		7	2339.43	9.82
	SS CC GL		8	2341.35	11.74

* Indicates no adjustment terms were selected by AIC.

**Fig 5:**
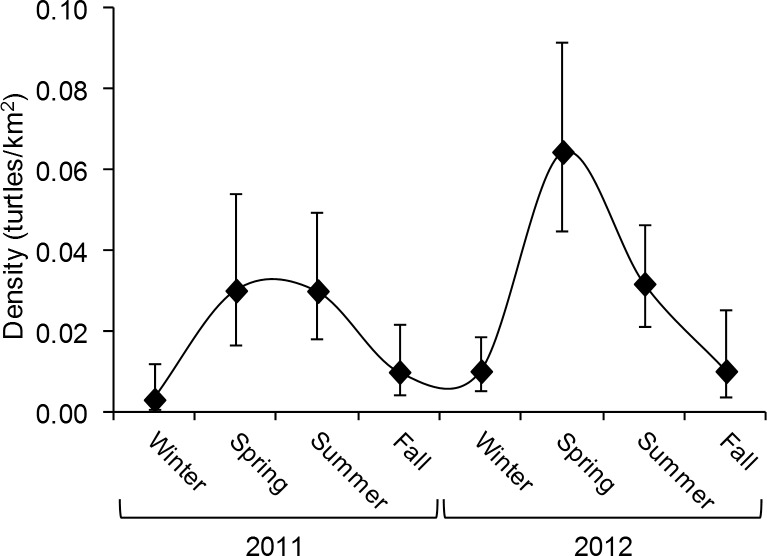
Seasonal trend in sea turtle surface density with 95% confidence intervals estimated from conventional distance sampling analysis of aerial surveys. Turtle densities varied with season in both years. The large peak in spring 2012 corresponded with a larger than normal number of nesting loggerhead turtles.

Estimates of sea turtle density were generally higher for spring (March-May) and summer (June-August) seasons than during fall (September-November) and winter (December-February) in both years (Fig 5). Additionally, estimates for spring 2012 were considerably higher than those for any other season (Table 1).

## Discussion

Aerial surveys are useful for assessing large, highly mobile marine vertebrates that must surface to breathe, such as sea turtles, and provide valuable information on the spatial and temporal shifts in distributions over large areas. However, a key shortcoming of many aerial surveys is relatively restricted temporal coverage, which limits the range of hypotheses that can be tested using these data. Our two-year long, monthly surveys provided a unique temporal perspective on sea turtle habitat use across seasons and in both a major warm water current and adjacent coastal waters. Such data have utility because they highlight spatially and temporally specific shifts in the abundance of these marine fauna. Our study represents the first estimation of the variation in sea turtle density and abundance in the waters offshore of the eastern coast of Florida at the seasonal level and provides a much-needed assessment of annual variation in sea turtle abundance coincident with critical breeding and nesting habitat.

The most frequently observed species, loggerhead (*C*. *caretta*) and green turtle (*C*. *mydas*), demonstrated similar spatial distributions within our study area (Fig 3) and correspond with the most abundant species nesting in this area [28]. However, loggerhead sightings increased in spring while green turtle sightings increased markedly in summer (Fig 3). The much smaller numbers of leatherbacks (*D*. *coriacea*) and Kemp’s ridleys (*L*. *kempii*) made it impossible to draw any conclusions on their spatial distributions. Additionally, no hawksbills (*E*. *imbricata*) were observed during our surveys. This may be due to morphological similarities between hawksbill and green sea turtles. Without a positive identification, any observed hawksbills likely were classified as unidentified cheloniids. Loggerhead and green turtles were observed on every survey conducted during our study, demonstrating their presence in these waters year-round and their potential for interaction with any ocean energy technologies that may be placed in this area.

Turtles also displayed a strong affinity for more coastal waters with 72.8% of all observations west of the approximate core of the Florida Current (~20 km offshore). Previous studies show that sea turtles associate with oceanic currents [21,41–44] and post-hatchling loggerhead turtles are carried by the Florida Current to the Gulf Stream into the North Atlantic Gyre [31]. It is assumed that sea turtles of various life stages and all species that hatch in Florida encounter the Florida Current. Our results clearly indicate the presence of these threatened and endangered species in the southeast portion of the Florida Current and its surrounding waters (Fig 5), demonstrating the need for improved assessments of their in-water distributions and movements to more fully understand any impacts of future ocean energy developments.

There were seasonal variations of density and abundance estimates prevalent throughout our study period; higher estimates were during the spring and summer surveys and lower estimates during the fall and winter surveys (Fig 5). The coastal area of South Florida adjacent to our study hosts one of the world’s largest loggerhead rookeries [24, 25] and increasing green turtle [18] and leatherback breeding populations [17], so it is reasonable to conclude that breeding and nesting turtles made up a large portion of our sightings during spring and summer (nesting season). Additionally, size classes recorded during surveys indicated larger individuals were identified more often during spring and summer surveys (n = 52) than winter and fall surveys (n = 5). Non-breeding turtles also migrate through Florida’s waters en route to northern feeding grounds in the spring and summer months, returning to more southern habitats in the winter and fall months [11, 15]. These breeding and foraging migrations likely hold a large influence over the seasonal shifts in density and abundance observed in this study.

Additionally, the inter-annual variation of our density estimates highlights the potentially large variability in sea turtle behavior. Changes of the magnitudes observed over the two years can complicate accurate estimation of their spatial and temporal densities. Our estimate of density from spring 2012 was more than twice that from spring 2011 (Table 1, Fig 5). However, there is independent evidence that the trends in density between the two seasons likely are representative; the 2012 nest counts for loggerheads were exceptionally high as compared with 2011 suggesting more turtles were nesting since nests per females is constrained [27]. Thus fluctuations in nesting behavior from year to year should be considered in interpreting results of surveys such as the one described here because such temporal shifts in local abundance impact estimations of sea turtle presence. Our study highlights the need for long term temporal monitoring of in-water sea turtle distributions and movements to examine how density and abundance vary across years.

This study clearly demonstrates the value of aerial surveys in (i) identifying the spatial distributions of marine turtles (protected species) in areas where there is potential for interactions with industry developments and (ii) estimating seasonal changes in density in ways that may be considered the effective management of species.

Despite the achievements of this study, there were several limitations. Selection of the conventional distance sampling (CDS) model over the multiple covariate distance sampling (MCDS) models was likely due to survey design and selection of flight dates for optimal surveying conditions. Because surveys were only conducted in optimal conditions, we effectively minimized the influence sea state and weather conditions that were considered in our MCDS analyses. Hence, inclusion of sea state and weather covariates did not improve the model of the detection function and thereby did not improve estimates of density or abundance. Although this did provide the benefit of removing potential sources of perception bias, it precluded the use of the more rigorous multiple covariate distance sampling methods with our observations.

Our density and abundance estimates are underestimates because they are surface estimates and are not corrected for perception bias or availability bias. Perception bias, animals at the surface that were not detected by observers, could not be addressed because equipment limitations prevented using a “double-platform” approach [59]. Availability bias, presence of animals underwater that cannot be detected by the observers, can be corrected for using concurrent dive profiles to assess the percentage of time spent at the surface. However, the scope and budget of the study prevented simultaneous tracking of turtles with time depth devices during the aerial surveys. Further, variation in dive duration correction is complex and can vary greatly with species, age, and oceanographic factors such as sea surface temperature or depth [62]. Nevertheless, a recent study on the effects of availability and perception bias on the results of aerial surveys for marine turtles in the Torres Strait indicates that uncorrected estimates are likely 20 times lower than estimates corrected for these biases [63]. This further illustrates the importance of this body of water for sea turtle species. Though, this estimate is predominantly based on adjustments for availability bias, it highlights the need for dedicated research that includes bias correction of aerial survey data to provide more robust density and abundance estimates. Finally, sea turtle distributions have been associated with a variety of oceanographic features including sea surface temperature [11, 44, 64], ocean depth or bathymetry [10, 65–67], chlorophyll a concentrations [64, 67], and mesoscale eddies [41], which should also be considered in future studies of sea turtle distributions.

Estimates of density and abundance are of particular interest for management and development of new conservation strategies for imperiled species like sea turtles because they provide valuable baselines for comparisons of changes in habitat uses or marine management outcomes. Aerial survey data for marine turtles have influenced fisheries management, habitat use and protection policies, and water use at local and large spatial scales [48–50, 55]. In our study area, the Florida Current is of particular interest because it has the potential to provide an alternative renewable energy source that may be captured by a variety of kinetic energy current capture technologies [68]. The combination of existing submerged land leases for energy development, increased interests in developing current capture technologies, and ongoing research on implementation of these technologies necessitates a clear understanding of the potential effects of ocean energy development on sea turtles and other marine life. Establishing these baseline abundance estimates and continuing research to improve our understanding of sea turtle distributions and movements in these waters will aid in assessing the potential for interaction with energy capturing or other interests that share these important waters used by sea turtles.

## Supporting Information

S1 Dataset Dataset of sea turtle sightings from aerial surveys in 2011 and 2012 used in distance sampling analyses to estimate density and abundance. (PDF)
