# Supplementary material for: Seasonal Variation in Sea Turtle Density and Abundance in the Southeast Florida Current and Surrounding Waters
Source: PLoS One. 2015 Dec 30;10(12):e0145980. doi: 10.1371/journal.pone.0145980 (PMC4696830; doi:10.1371/journal.pone.0145980)
Supplement: S1 Dataset — (PDF) [file pone.0145980.s001.pdf]

Full Dataset

Season ID	Label	Area	Transect_ID	Line_Transect _Label	Line length	Transect _No.	Cloud Cover	Glare	Sea State	Sighting _ID	Perpendicular distance	Species	Size	Sea State	Observer
1	Winter	13000	1	110128_01	84.2	1	MC	G	3						
1	Winter	13000	2	110128_02	92.6	2	MC	F	3						
1	Winter	13000	3	110129_03	53.71	3	SU	F	3						
1	Winter	13000	4	110129_04	52.5	4	SU	F	3						
1	Winter	13000	5	110129_05	84.26	5	SU	F	2						
1	Winter	13000	6	110129_06	54.21	6	MS	F	2						
1	Winter	13000	7	110129_07	50	7	MS	F	2						
1	Winter	13000	8	110129_08	50	8	MS	F	2						
1	Winter	13000	9	110129_09	79.64	9	MS	F	2						
1	Winter	13000	10	110129_10	79.64	10	MS	F	2						
1	Winter	13000	11	110129_11	50	11	MS	F	2						
1	Winter	13000	12	110129_12	50	12	PC	F	2						
1	Winter	13000	13	110226_01	91.2	1	PC	F							
1	Winter	13000	14	110226_02	92.6	2	PC	F		1	259.4	U	U	0	EM
1	Winter	13000	15	110226_03	52.49	3	MS	F							
1	Winter	13000	16	110226_04	54.35	4	MS	F							
1	Winter	13000	17	110226_05	86.67	5	MS	F							
1	Winter	13000	18	110226_06	83.69	6	SU	F							
1	Winter	13000	19	110226_07	50	7	MS	F							
1	Winter	13000	20	110226_08	50	8	MS	F							
1	Winter	13000	21	110226_09	83.42	9	MS	F							
1	Winter	13000	22	110226_10	79.64	10	MS	F							
1	Winter	13000	23	110226_11	50	11	MS	F							
1	Winter	13000	24	110226_12	50	12	MS	F							
1	Winter	13000	25	111216_01	96.17	1	PC	F							
1	Winter	13000	26	111216_02	104.03	2	PC	F							
1	Winter	13000	27	111216_03	56	3	PC	F		2	86.81	Cc	M	1	SEC
1	Winter	13000	27	111216_03	56	3	PC	F		3	86.81	Cc	M	1	SEC
1	Winter	13000	28	111216_04	56.35	4	PC	F							
1	Winter	13000	29	111216_05	77.53	5	PC	F							
1	Winter	13000	30	111216_06	77.66	6	PC	F							
1	Winter	13000	31	111216_07	60.74	7	PC	F							
1	Winter	13000	32	111216_08	52.37	8	PC	F							
1	Winter	13000	33	111216_09	89.1	9	PC	F							
1	Winter	13000	34	111216_10	95.06	10	PC	F							
1	Winter	13000	35	111216_11	56.51	11	PC	F							
1	Winter	13000	36	111216_12	54.78	12	PC	F							

1	Winter	13000	37	120301_01	35.13	1	PC	F							
1	Winter	13000	38	120301_02	35.99	2	PC	F							
1	Winter	13000	39	120301_03	105.86	3	PC	F							
1	Winter	13000	40	120301_04	107.85	4	PC	F		4	50.13	U	S	1	CB
1	Winter	13000	41	120301_05	41.08	5	PC	F							
1	Winter	13000	42	120301_06	41.15	6	PC	F		5	75.4	Cc	S	1	CB
1	Winter	13000	43	120301_07	102.06	7	MS	F							
1	Winter	13000	44	120301_08	102.63	8	PC	F							
1	Winter	13000	45	120301_09	40.48	9	MS	F							
1	Winter	13000	46	120301_10	32.8	10	PC	F							
1	Winter	13000	47	120301_11	33.79	11	PC	F		6	54.86	Cc	M	1	JP
1	Winter	13000	48	120301_12	45.69	12	MS	F		7	36.76	Cc	M	1	CB
1	Winter	13000	48	120301_12	45.69	12	MS	F		8	16.71	Cc	S	1	CB
1	Winter	13000	49	120301_13	104.71	13	PC	F		9	12.98	Cc	L	1	CB
1	Winter	13000	49	120301_13	104.71	13	PC	F		10	36.76	Cm	S	1	CB
1	Winter	13000	50	120301_14	103.8	14	PC	F		11	112.55	U	S	1	CB
1	Winter	13000	51	120301_15	38.73	15	MS	F							
1	Winter	13000	52	120301_16	42.63	16	MS	F							
1	Winter	13000	53	121216_01	36.95	1	MS	F		12	70	Cm	S	2	CB
1	Winter	13000	54	121216_02	38.2	2	MS	F							
1	Winter	13000	55	121216_03	105.96	3	MS	F							
1	Winter	13000	56	121216_04	108.33	4	PC	F							
1	Winter	13000	57	121216_05	42.96	5	MC	F							
1	Winter	13000	58	121216_06	42.61	6	PC	F							
1	Winter	13000	59	121216_07	102.23	7	MC	F		13	161.25	Cm	M	2	CB
1	Winter	13000	60	121216_08	99.08	8	MC	F							
1	Winter	13000	61	121216_09	39.01	9	SU	F							
1	Winter	13000	62	121216_10	35.09	10	MS	F							
1	Winter	13000	63	121216_11	35.36	11	MS	F		14	75.4	Cm	L	2	CB
1	Winter	13000	64	121216_12	43.3	12	MS	F							
1	Winter	13000	65	121216_13	105.7	13	MS	F							
1	Winter	13000	66	121216_14	104.98	14	MS	F							
1	Winter	13000	67	121216_15	37.67	15	SU	F							
1	Winter	13000	68	121216_16	43.93	16	MS	F							
1	Winter	13000	69	130116_01	36.95	1	MS	F							
1	Winter	13000	70	130116_02	38.56	2	MS	F							
1	Winter	13000	71	130116_03	106.93	3	MS	F							
1	Winter	13000	72	130116_04	107.41	4	MS	F							
1	Winter	13000	73	130116_05	43.04	5	PC	G	1						
1	Winter	13000	74	130116_06	43.63	6	PC	G	2						

1	Winter	13000	75	130116_07	104.19	7	PC	G	1						
1	Winter	13000	76	130116_08	98.61	8	PC	G	2						
1	Winter	13000	77	130116_09	40.92	9	PC	G	2						
1	Winter	13000	78	130116_10	36.07	10	PC	G	2						
1	Winter	13000	79	130116_11	37.12	11	PC	G	1						
1	Winter	13000	80	130116_12	44.33	12	PC	G	1						
1	Winter	13000	81	130116_13	105.82	13	PC	G	1						
1	Winter	13000	82	130116_14	104.89	14	PC	G	1						
1	Winter	13000	83	130116_15	41.39	15	PC	G	1						
1	Winter	13000	84	130116_16	44.88	16	PC	G	1	15	112.55	Cm	S	0	CB
2	Spring	13000	85	110325_01	90.16	1	PC	G	0						
2	Spring	13000	86	110325_02	102.79	2	PC	G	0						
2	Spring	13000	87	110325_03	55.65	3	PC	G	0						
2	Spring	13000	88	110325_04	58.21	4	PC	G	0						
2	Spring	13000	89	110325_05	78.65	5	PC	G	0						
2	Spring	13000	90	110325_06	76.59	6	PC	G	0						
2	Spring	13000	91	110325_07	59.66	7	PC	G	0						
2	Spring	13000	92	110325_08	53.75	8	PC	G	0						
2	Spring	13000	93	110325_09	90.12	9	PC	G	0						
2	Spring	13000	94	110325_10	96.01	10	PC	G	0						
2	Spring	13000	95	110325_11	56.18	11	MS	G	0						
2	Spring	13000	96	110325_12	57.81	12	PC	G	0						
2	Spring	13000	97	110507_01	86.49	1	PC	G	0						
2	Spring	13000	98	110507_02	86.49	2	PC	G	0						
2	Spring	13000	99	110507_03	57.95	3	PC	G	0	16	99.15	Cc	U	1	EM
2	Spring	13000	100	110507_04	56.84	4	PC	G	0	17	70	Cc	M	2	EM
2	Spring	13000	101	110507_05	76.85	5	MS	G	0	18	259.4	U	M	1	SEC
2	Spring	13000	101	110507_05	76.85	5	MS	G	0	19	335.62	Cc	U	2	EM
2	Spring	13000	101	110507_05	76.85	5	MS	G	0	20	203.87	U	M	2	SEC
2	Spring	13000	102	110507_06	76.12	6	MS	G	0	21	313.97	U	S	2	EM
2	Spring	13000	102	110507_06	76.12	6	MS	G	0	22	70	U	M	2	SEC
2	Spring	13000	102	110507_06	76.12	6	MS	G	0	23	99.15	U	M	2	SEC
2	Spring	13000	102	110507_06	76.12	6	MS	G	0	24	170.97	U	M	2	SEC
2	Spring	13000	103	110507_07	59.23	7	SU	F	0	25	135.08	U	M-L	2	SEC
2	Spring	13000	103	110507_07	59.23	7	SU	F	0	26	181.27	Cc	M-L	2	SEC
2	Spring	13000	103	110507_07	59.23	7	SU	F	0	27	99.15	U	M-L	2	SEC
2	Spring	13000	103	110507_07	59.23	7	SU	F	0	28	294.21	U	U	2	EM
2	Spring	13000	103	110507_07	59.23	7	SU	F	0	29	99.15	U	L	2	SEC
2	Spring	13000	104	110507_08	55.05	8	MS	G	0	30	70	Cc	L	2	SEC
2	Spring	13000	104	110507_08	55.05	8	MS	G	0	31	243.97	U	U	2	EM

2	Spring	13000	104	110507_08	55.05	8	MS	G	0	32	135.08	Cc	M	2	SEC
2	Spring	13000	104	110507_08	55.05	8	MS	G	0	33	70	Cc	M-L	2	SEC
2	Spring	13000	105	110507_09	92.33	9	SU	G	0						
2	Spring	13000	106	110507_10	98.61	10	PC	F	0	34	335.62	U	M	2	SEC
2	Spring	13000	107	110507_11	57.43	11	SU	G	1	35	112.55	U	U	1	SEC
2	Spring	13000	107	110507_11	57.43	11	SU	G	1	36	70	Cc	M	1	SEC
2	Spring	13000	107	110507_11	57.43	11	SU	G	1	37	243.97	U	M	1	SEC
2	Spring	13000	108	110507_12	55.33	12	PC	F	1						
2	Spring	13000	109	110527_01	97.96	1	PC	G	1	38	294.21	Cm	M	1	JP
2	Spring	13000	110	110527_02	101.91	2	PC	G	1						
2	Spring	13000	111	110527_03	57.27	3	PC	G	1						
2	Spring	13000	112	110527_04	57.63	4	PC	G	1	39	127.21	Cm	M-L	1	JP
2	Spring	13000	112	110527_04	57.63	4	PC	G	1	40	216.32	Cm	L	1	JP
2	Spring	13000	113	110527_05	78.2	5	PC	G	1						
2	Spring	13000	114	110527_06	78.15	6	PC	G	1	41	143.35	U	M-L	1	JP
2	Spring	13000	115	110527_07	59.86	7	MC	F	1	42	119.71	Cc	L	2	JP
2	Spring	13000	115	110527_07	59.86	7	MC	F	1	43	5.76	Cm	M-L	2	JP
2	Spring	13000	116	110527_08	55.05	8	MC	G	1						
2	Spring	13000	117	110527_09	80.56	9	MC	F	1						
2	Spring	13000	118	110527_10	84.27	10	MC	G	1						
2	Spring	13000	119	110527_11	57.22	11	MC	G	1						
2	Spring	13000	120	110527_12	55.86	12	MC	F	1	44	135.08	Cm	S-M	2	JP
2	Spring	13000	120	110527_12	55.86	12	MC	F	1	45	92.86	Cc	M-L	2	JP
2	Spring	13000	121	120324_01	35.68	1	PC	G	1						
2	Spring	13000	122	120324_02	37.54	2	PC	G	1	46	86.81	Cm	S	0	CB
2	Spring	13000	123	120324_03	107.14	3	MS	G	1						
2	Spring	13000	124	120324_04	109.2	4	PC	G	1						
2	Spring	13000	125	120324_05	41.59	5	PC	G	1						
2	Spring	13000	126	120324_06	43.34	6	PC	F	1						
2	Spring	13000	127	120324_07	102.56	7	MS	G	1						
2	Spring	13000	128	120324_08	104.46	8	MS	F	1						
2	Spring	13000	129	120324_09	41.88	9	PC	F	1						
2	Spring	13000	130	120324_10	33.19	10	MS	G	1	47	161.25	U	M	0	CB
2	Spring	13000	131	120324_11	33.91	11	MS	G	1						
2	Spring	13000	132	120324_12	34.22	12	MS	G	1						
2	Spring	13000	133	120324_13	103.53	13	MC	G	2	48	86.81	Cm	M	0	SEC
2	Spring	13000	133	120324_13	103.53	13	MC	G	2	49	143.35	Cc	M	0	SEC
2	Spring	13000	133	120324_13	103.53	13	MC	G	2	50	54.86	Cc	L	0	CB
2	Spring	13000	134	120324_14	101.74	14	MS	G	1	51	192.22	Cc	S	0	SEC
2	Spring	13000	134	120324_14	101.74	14	MS	G	1	52	54.86	Cc	M	1	SEC

2	Spring	13000	135	120324_15	38.71	15	PC	G	1	53	50.13	Cm	M	0	CB
2	Spring	13000	135	120324_15	38.71	15	PC	G	1	54	229.65	Cc	S	0	SEC
2	Spring	13000	136	120324_16	41.9	16	PC	G	1						
2	Spring	13000	137	120505_01	35.67	1	MC	G	1						
2	Spring	13000	138	120505_02	38.58	2	PC	G	1	55	99.15	Lk	S	1	CB
2	Spring	13000	139	120505_03	106.56	3	PC	G	1	56	170.97	Cm	M	1	CB
2	Spring	13000	140	120505_04	108.65	4	PC	G	1	57	99.15	Cc	L	1	SEC
2	Spring	13000	140	120505_04	108.65	4	PC	G	1	58	9.33	Cc	L	1	CB
2	Spring	13000	140	120505_04	108.65	4	PC	G	1	59	112.55	Cc	M	1	CB
2	Spring	13000	141	120505_05	42.4	5	PC	G	1	60	119.71	U	S-M	1	SEC
2	Spring	13000	141	120505_05	42.4	5	PC	G	1	61	119.71	U	S-M	1	SEC
2	Spring	13000	141	120505_05	42.4	5	PC	G	1	62	135.08	U	M	1	SEC
2	Spring	13000	141	120505_05	42.4	5	PC	G	1	63	59.74	Cc	L	1	CB
2	Spring	13000	141	120505_05	42.4	5	PC	G	1	64	75.4	Cc	L	1	CB
2	Spring	13000	141	120505_05	42.4	5	PC	G	1	65	75.4	Cc	L	1	CB
2	Spring	13000	141	120505_05	42.4	5	PC	G	1	66	135.08	Cc	M	1	SEC
2	Spring	13000	142	120505_06	44.02	6	PC	G	1	67	70	Cc	L	1	SEC
2	Spring	13000	142	120505_06	44.02	6	PC	G	1	68	119.71	Cc	M	1	CB
2	Spring	13000	143	120505_07	103.24	7	PC	G	1	69	28.43	Cc	M	1	CB
2	Spring	13000	143	120505_07	103.24	7	PC	G	1	70	112.55	Cc	L	1	SEC
2	Spring	13000	143	120505_07	103.24	7	PC	G	1	71	243.97	Cc	L	1	SEC
2	Spring	13000	144	120505_08	105.41	8	PC	G	1	72	112.55	Cc	L	1	SEC
2	Spring	13000	145	120505_09	40.58	9	PC	G	1	73	45.54	Cc	L	1	SEC
2	Spring	13000	145	120505_09	40.58	9	PC	G	1	74	99.15	Cc	L	1	SEC
2	Spring	13000	146	120505_10	34.04	10	PC	G	1						
2	Spring	13000	147	120505_11	35.09	11	PC	G	1						
2	Spring	13000	148	120505_12	43.5	12	PC	G	1	75	216.32	Cc	M	1	CB
2	Spring	13000	149	120505_13	105.03	13	PC	G	1	76	24.43	Cc	M	1	CB
2	Spring	13000	149	120505_13	105.03	13	PC	G	1	77	24.43	Cc	L	1	SEC
2	Spring	13000	149	120505_13	105.03	13	PC	G	1	78	161.25	Cc	S	1	CB
2	Spring	13000	150	120505_14	104.54	14	PC	G	1	79	119.71	Cc	M	1	CB
2	Spring	13000	151	120505_15	39.73	15	PC	F	1	80	216.32	Cc	M	1	CB
2	Spring	13000	151	120505_15	39.73	15	PC	F	1	81	243.97	U	M	1	SEC
2	Spring	13000	152	120505_16	43.95	16	PC	G	1	82	119.71	Cc	M	1	CB
2	Spring	13000	152	120505_16	43.95	16	PC	G	1	83	181.27	U		1	SEC
2	Spring	13000	152	120505_16	43.95	16	PC	G	1	84	135.08	Cc		1	CB
2	Spring	13000	152	120505_16	43.95	16	PC	G	1	85	135.08	Cc		1	CB
2	Spring	13000	152	120505_16	43.95	16	PC	G	1	86	45.54	Cc		1	SEC
2	Spring	13000	153	120519_03	71.14	3	MS	G	1						
2	Spring	13000	154	120519_04	107.68	4	PC	G	1	87	41.09	Cc	M	2	CB

2	Spring	13000	154	120519_04	107.68	4	PC	G	1	88	50.13	Cc	S	1	SEC
2	Spring	13000	155	120519_05	79.77	5	MS	G	1	89	45.54	Cc	M	0	SEC
2	Spring	13000	155	120519_05	79.77	5	MS	G	1	90	12.98	Cc	M	0	SEC
2	Spring	13000	156	120519_06	82.74	6	PC	G	1						
2	Spring	13000	157	120519_07	60.45	7	PC	G	1	91	12.98	Cc	M	1	SEC
2	Spring	13000	157	120519_07	60.45	7	PC	G	1	92	75.4	Cc	S	0	SEC
2	Spring	13000	158	120519_08	63.08	8	SU	G	1	93	119.71	Cm	M	1	CB
2	Spring	13000	159	120519_09	40.38	9	SU	G	1	94	203.87	Dc	L	0	CB
2	Spring	13000	159	120519_09	40.38	9	SU	G	1	95	161.25	Cc	M	0	CB
2	Spring	13000	159	120519_09	40.38	9	SU	G	1	96	192.22	Cc	M	0	SEC
2	Spring	13000	159	120519_09	40.38	9	SU	G	1	97	41.09	Cc	L	0	CB
2	Spring	13000	160	120519_10	36.02	10	SU	G	1	98	12.98	Cc	L	0	CB
2	Spring	13000	160	120519_10	36.02	10	SU	G	1	99	127.21	Cc	S	0	SEC
2	Spring	13000	161	120519_11	35.33	11	SU	G	0	100	70	Cc	M	0	CB
2	Spring	13000	162	120519_12	42.85	12	MS	G	0	101	28.43	Cc	S	0	CB
2	Spring	13000	163	120519_13	105.66	13	SU	G	0	102	75.4	Cc	S	0	SEC
2	Spring	13000	163	120519_13	105.66	13	SU	G	0	103	119.71	Cc	S	0	SEC
2	Spring	13000	163	120519_13	105.66	13	SU	G	0	104	41.09	Cc	M	0	SEC
2	Spring	13000	163	120519_13	105.66	13	SU	G	0	105	20.53	Cm	M	0	SEC
2	Spring	13000	163	120519_13	105.66	13	SU	G	0	106	36.76	Cm	L	0	SEC
2	Spring	13000	163	120519_13	105.66	13	SU	G	0	107	20.53	Cc	S	0	CB
2	Spring	13000	164	120519_14	105.05	14	SU	G	1	108	12.98	Cc	S	0	CB
2	Spring	13000	164	120519_14	105.05	14	SU	G	1	109	32.54	Cc	S	0	SEC
2	Spring	13000	164	120519_14	105.05	14	SU	G	1	110	54.86	Cc	S	0	SEC
2	Spring	13000	164	120519_14	105.05	14	SU	G	1	111	64.78	Cc	S	0	SEC
2	Spring	13000	164	120519_14	105.05	14	SU	G	1	112	181.27	Cc	L	0	SEC
2	Spring	13000	165	120519_15	41.95	15	MS	G	0	113	161.25	Cm	M	0	SEC
2	Spring	13000	165	120519_15	41.95	15	MS	G	0	114	54.86	Cc		0	SEC
2	Spring	13000	166	120519_16	44.09	16	MS	G	0	115	75.4	Cm	M	0	CB
2	Spring	13000	166	120519_16	44.09	16	MS	G	0	116	59.74	Cc	M	0	CB
2	Spring	13000	166	120519_16	44.09	16	MS	G	0	117	86.81	Cc	M	0	SEC
2	Spring	13000	166	120519_16	44.09	16	MS	G	0	118	54.86	Cc	M	0	CB
3	Summer	13000	167	110617_01	96.03	1	MS	G	0						
3	Summer	13000	168	110617_02	103.4	2	PC	G	1	119	161.25	Cm	M-L	1	JP
3	Summer	13000	168	110617_02	103.4	2	PC	G	1	120	99.15	U	S	1	JP
3	Summer	13000	168	110617_02	103.4	2	PC	G	1	121	276.09	U	U	1	JP
3	Summer	13000	169	110617_03	57.12	3	MS	G	0	122	143.35	Cc	U	1	JP
3	Summer	13000	169	110617_03	57.12	3	MS	G	0	123	135.08	Cc	L	1	JP
3	Summer	13000	169	110617_03	57.12	3	MS	G	0	124	181.27	Cm	M-L	1	JP
3	Summer	13000	169	110617_03	57.12	3	MS	G	0	125	64.78	U	S	1	JP

3	Summer	13000	170	110617_04	58.61	4	PC	G	0	126	45.54	Cc	L	1	JP
3	Summer	13000	170	110617_04	58.61	4	PC	G	0	127	152.06	U	M	1	JP
3	Summer	13000	170	110617_04	58.61	4	PC	G	0	128	143.35	U	M	1	JP
3	Summer	13000	170	110617_04	58.61	4	PC	G	0	129	135.08	U	U	1	EM
3	Summer	13000	171	110617_05	75.85	5	PC	G	0	130	5.76	Cm	S-M	1	JP
3	Summer	13000	171	110617_05	75.85	5	PC	G	0	131	64.78	Cm	L	1	JP
3	Summer	13000	172	110617_06	78.55	6	PC	G	0	132	294.21	U	S-M	1	JP
3	Summer	13000	172	110617_06	78.55	6	PC	G	0	133	170.97	Cm	M-L	1	JP
3	Summer	13000	173	110617_07	58.71	7	MS	G	0	134	32.54	Cm	M	1	JP
3	Summer	13000	173	110617_07	58.71	7	MS	G	0	135	152.06	Cm	M	1	JP
3	Summer	13000	174	110617_08	56.53	8	MS	G	0						
3	Summer	13000	175	110617_09	90.14	9	MS	G	0						
3	Summer	13000	176	110617_10	96.38	10	PC	G	0	136	203.87	Cm	L	1	JP
3	Summer	13000	177	110617_11	29.89	11	PC	G	1						
3	Summer	13000	178	110617_12	54.78	12	PC	G	1						
3	Summer	13000	179	110715_01	86.49	1	PC	G	1						
3	Summer	13000	180	110715_02	103.4	2	PC	G	1	137	119.71	Cc	L	1	JP
3	Summer	13000	180	110715_02	103.4	2	PC	G	1	138	50.13	Cc	M	2	SEC
3	Summer	13000	181	110715_03	57.12	3	PC	F	1	139	86.81	Cc	L	1	SEC
3	Summer	13000	181	110715_03	57.12	3	PC	F	1	140	105.7	Cc	M	1	SEC
3	Summer	13000	181	110715_03	57.12	3	PC	F	1	141	99.15	Cm	n/a	1	JP
3	Summer	13000	182	110715_04	58.61	4	PC	G	1	142	75.4	Cm	S	1	JP
3	Summer	13000	183	110715_05	75.85	5	PC	G	1	143	45.54	Cm	M	1	SEC
3	Summer	13000	184	110715_06	78.55	6	PC	G	1	144	119.71	Cm	M	1	JP
3	Summer	13000	185	110715_07	58.71	7	PC	G	1						
3	Summer	13000	186	110715_08	56.53	8	PC	G	1						
3	Summer	13000	187	110715_09	89.73	9	PC	G	1						
3	Summer	13000	188	110715_10	97.46	10	PC	G	1						
3	Summer	13000	189	110715_11	49.82	11	SU	G	1						
3	Summer	13000	190	110715_12	54.78	12	SU	G	1	145	70	U	M	1	SEC
3	Summer	13000	191	110813_01	97.71	1	MS	G	1						
3	Summer	13000	192	110813_02	103.22	2	MS		1						
3	Summer	13000	193	110813_03	56.52	3	MC	F	1	146	143.35	Cm	M	0	JP
3	Summer	13000	194	110813_04	57.93	4	MC	G	2						
3	Summer	13000	195	110813_05	77.78	5	MC	F	1	147	64.78	U	M	1	SEC
3	Summer	13000	196	110813_06	78.33	6	MC	G	1						
3	Summer	13000	197	110813_07	59.58	7	MC	F	1						
3	Summer	13000	198	110813_08	53.06	8	PC	F	1	148	81	Cc	L	1	SEC
3	Summer	13000	199	110813_09	90.05	9	PC	G	1						
3	Summer	13000	200	110813_10	97.26	10	PC	G	0						

3	Summer	13000	201	110813_11	56.89	11	PC	F	0						
3	Summer	13000	202	110813_12	57.07	12	PC	F	0						
3	Summer	13000	203	120701_01	33.68	1	PC	G	0						
3	Summer	13000	204	120701_02	38.72	2	PC	F	0						
3	Summer	13000	205	120701_03	106.09	3	PC	G	0	149	359.44	Dc	L	0	CB
3	Summer	13000	206	120701_04	107.56	4	PC	G	0	150	64.78	Cm	M	0	CB
3	Summer	13000	207	120701_05	42.62	5	SU	F	1	151	243.97	U	S	0	CB
3	Summer	13000	208	120701_06	44.56	6	SU	G	1	152	259.4	Cm	M	1	SEC
3	Summer	13000	209	120701_07	102.86	7	SU	F	0	153	203.87	Cc	M	0	SEC
3	Summer	13000	210	120701_08	102.79	8	SU	G	0	154	16.71	Cm	L	0	CB
3	Summer	13000	211	120701_09	31.97	9	SU	G	0	155	135.08	Cm	M	0	CB
3	Summer	13000	211	120701_09	31.97	9	SU	G	0	156	294.21	Cm	M	0	SEC
3	Summer	13000	212	120701_10	34.38	10	SU	G	1						
3	Summer	13000	213	120701_11	36.19	11	SU	G	0						
3	Summer	13000	214	120701_12	43.93	12	MS	G	1						
3	Summer	13000	215	120701_13	105.29	13	MS	F	0						
3	Summer	13000	216	120701_14	102.51	14	MS	G	0	157	70	Cm	M/L	1	SEC
3	Summer	13000	217	120701_15	40.67	15	MS	G	1	158	32.54	Cm	M	1	CB
3	Summer	13000	218	120701_16	41.47	16	MS	G	0	159	75.4	U	L	0	CB
3	Summer	13000	219	120728_01	31.47	1	MS	F	1	160	36.76	Cm	M	0	CB
3	Summer	13000	220	120728_02	37.72	2	MS	G	0						
3	Summer	13000	221	120728_03	106.55	3	MS	G	1	161	81	Cm	M	0	SEC
3	Summer	13000	221	120728_03	106.55	3	MS	G	1	162	70	Cc	L	0	CB
3	Summer	13000	222	120728_04	108.42	4	MS	F	0						
3	Summer	13000	223	120728_05	42.23	5	PC	G	0	163	20.53	U	S	0	CB
3	Summer	13000	223	120728_05	42.23	5	PC	G	0	164	75.4	Cm	M	0	CB
3	Summer	13000	224	120728_06	42.62	6	PC	G	0	165	64.78	Cc	S	0	SEC
3	Summer	13000	224	120728_06	42.62	6	PC	G	0	166	12.98	Cc	L	0	CB
3	Summer	13000	225	120728_07	102.52	7	SU	F	0						
3	Summer	13000	226	120728_08	98.31	8	SU	G	0						
3	Summer	13000	227	120728_09	39.93	9	PC	G	0	167	229.65	Cc	M	0	CB
3	Summer	13000	227	120728_09	39.93	9	PC	G	0	168	112.55	Cc	M	0	CB
3	Summer	13000	227	120728_09	39.93	9	PC	G	0	169	192.22	Cm	M	0	SEC
3	Summer	13000	227	120728_09	39.93	9	PC	G	0	170	203.87	U	M	0	SEC
3	Summer	13000	228	120728_10	33.11	10	PC	G	0						
3	Summer	13000	229	120728_11	36.29	11	PC	F	0	171	192.22	Cm	M	1	SEC
3	Summer	13000	230	120728_12	42.82	12	PC	G	0						
3	Summer	13000	231	120728_13	104.68	13	PC	G	0	172	119.71	Cm	M	0	SEC
3	Summer	13000	232	120728_14	104.91	14	PC	G	0	173	75.4	U	S	0	SEC
3	Summer	13000	233	120728_15	40.4	15	PC	G	1	174	99.15	Cc	M	0	CB

3	Summer	13000	233	120728_15	40.4	15	PC	G	1	175	81	Cm	M	0	CB
3	Summer	13000	234	120728_16	42.08	16	PC	G	0						
3	Summer	13000	235	120811_01	34.01	1	PC	G	0						
3	Summer	13000	236	120811_02	36.83	2	SU	G	0	176	2.25	Cm	L	0	CB
3	Summer	13000	236	120811_02	36.83	2	SU	G	0	177	64.78	Cc	S	1	SEC
3	Summer	13000	237	120811_03	105.09	3	MS	G	0	178	143.35	Cm	M	0	CB
3	Summer	13000	237	120811_03	105.09	3	MS	G	0	179	143.35	Cc	S	1	SEC
3	Summer	13000	238	120811_04	107.04	4	SU	G	0	180	59.74	Cc	M	1	CB
3	Summer	13000	239	120811_05	40.31	5	MC	F	0						
3	Summer	13000	240	120811_06	41.16	6	MC	G	1	181	105.7	Cm	S	0	CB
3	Summer	13000	240	120811_06	41.16	6	MC	G	1	182	32.54	Cc	M	0	CB
3	Summer	13000	241	120811_07	101.97	7	MC	F	0	183	86.81	Cc	M	1	SEC
3	Summer	13000	241	120811_07	101.97	7	MC	F	0	184	70	Cc		1	SEC
3	Summer	13000	242	120811_08	98.52	8	MC	G	1						
3	Summer	13000	243	120811_09	40.84	9	PC	F	0						
3	Summer	13000	244	120811_10	35.19	10	MC	G	0	185	127.21	Cm	M	0	CB
3	Summer	13000	245	120811_11	35.73	11	PC	G	1						
3	Summer	13000	246	120811_12	41.82	12	MC	G	0						
3	Summer	13000	247	120811_13	104.11	13	MC	G	1						
3	Summer	13000	248	120811_14	103.47	14	MC	G	0						
3	Summer	13000	249	120811_15	38.82	15	MC	G	0						
3	Summer	13000	250	120811_16	41.99	16	PC	G	0						
4	Fall	13000	251	110916_01	98.12	1	MC	G	1						
4	Fall	13000	252	110916_02	103.78	2	PC	F	0	186	75.4	CM	M	0	SEC
4	Fall	13000	253	110916_03	56.4	3	MC	G	0						
4	Fall	13000	254	110916_04	58.06	4	PC	F	0						
4	Fall	13000	255	110916_05	76.86	5	PC	F	2	187	203.87	U	S	0	SEC
4	Fall	13000	255	110916_05	76.86	5	PC	F	2	188	75.4	Cm	L	0	JP
4	Fall	13000	256	110916_06	77.18	6	PC	F	2						
4	Fall	13000	257	110916_07	59.52	7	PC	G	1						
4	Fall	13000	258	110916_08	52.86	8	PC	G	1						
4	Fall	13000	259	110916_09	89.14	9	PC	G	1						
4	Fall	13000	260	110916_10	97.04	10	MS	G	1	189	203.87	U	S	0	SEC
4	Fall	13000	260	110916_10	97.04	10	MS	G	1	190	181.27	Dc		0	SEC
4	Fall	13000	261	110916_11	57.31	11	MS	G	0	191	24.43	U	S	0	SEC
4	Fall	13000	261	110916_11	57.31	11	MS	G	0	192	112.55	U	M	0	SEC
4	Fall	13000	262	110916_12	57.45	12	SU	G	1						
4	Fall	13000	263	111014_01	96.64	1	MS	F	0						
4	Fall	13000	264	111014_02	105.29	2	PC	F	1						
4	Fall	13000	265	111014_03	55.82	3	PC	B	1						

4	Fall	13000	266	111014_04	58.12	4	PC	F	1						
4	Fall	13000	267	111014_05	78.18	5	MS	G	0						
4	Fall	13000	268	111014_06	79.86	6	SU	F	0						
4	Fall	13000	269	111014_07	60.26	7	PC	G	1	193	135.08	Cc	M	1	SEC
4	Fall	13000	270	111014_08	54.13	8	MS	F	1						
4	Fall	13000	271	111014_09	49.2	9	MS	G	0						
4	Fall	13000	272	111014_10	96.72	10	PC	F	1						
4	Fall	13000	273	111014_11	56.84	11	PC	G	0						
4	Fall	13000	274	111014_12	56.88	12	MC	B	1						
4	Fall	13000	275	111123_01	98.89	1	PC	G	0						
4	Fall	13000	276	111123_02	103.73	2	PC	G	1						
4	Fall	13000	277	111123_03	54.18	3	PC	G	0						
4	Fall	13000	278	111123_04	57.98	4	PC	F	0						
4	Fall	13000	279	111123_05	78.1	5	PC	F	0						
4	Fall	13000	280	111123_06	78.88	6	PC	G	0						
4	Fall	13000	281	111123_07	60.07	7	PC	F	1						
4	Fall	13000	282	111123_08	52.56	8	PC	G	1						
4	Fall	13000	283	111123_09	86.62	9	PC	F	0						
4	Fall	13000	284	111123_10	96.56	10	PC	G	0						
4	Fall	13000	285	111123_11	56.66	11	PC	F	0						
4	Fall	13000	286	111123_12	56.61	12	PC	G	1	194	335.62	Cc	M	1	SEC
4	Fall	13000	286	111123_12	56.61	12	PC	G	1	195	181.27	Cc	M	1	SEC
4	Fall	13000	287	120901_01	34.5	1	SU	G	2						
4	Fall	13000	288	120901_02	39.71	2	SU	F	2						
4	Fall	13000	289	120901_03	105.98	3	SU	F	0	196	86.81	Cc	S	1	SEC
4	Fall	13000	289	120901_03	105.98	3	SU	F	0	197	32.54	Cc	L	1	CB
4	Fall	13000	290	120901_04	107.26	4	SU	G	1						
4	Fall	13000	291	120901_05	41.6	5	SU	G	0						
4	Fall	13000	292	120901_06	43.02	6	SU	G	1						
4	Fall	13000	293	120901_07	103.31	7	SU	G	0	198	152.06	Cm	M	0	CB
4	Fall	13000	293	120901_07	103.31	7	SU	G	0	199	70	Cm	L	0	SEC
4	Fall	13000	294	120901_08	104.5	8	SU	F	1						
4	Fall	13000	295	120901_09	39.41	9	SU	F	0						
4	Fall	13000	296	120901_10	35.81	10	SU	F	2						
4	Fall	13000	297	120901_11	36.15	11	SU	F	0						
4	Fall	13000	298	120901_12	42.77	12	SU	F	2						
4	Fall	13000	299	120901_13	104.41	13	SU	F	0						
4	Fall	13000	300	120901_14	103.61	14	SU	F	0						
4	Fall	13000	301	120901_15	39.52	15	SU	G	0						
4	Fall	13000	302	120901_16	42.5	16	SU	G	1						

4	Fall	13000	303	121019_01	34.54	1	MC	G	2						
4	Fall	13000	304	121019_02	37.35	2	PC	G	2						
4	Fall	13000	305	121019_03	106.77	3	MC	G	3	200	32.54	Cc	M	1	CB
4	Fall	13000	306	121019_04	105.57	4	PC	G	2						
4	Fall	13000	307	121019_05	41.87	5	MC	G	3						
4	Fall	13000	308	121019_06	43.46	6	PC	G	2						
4	Fall	13000	309	121019_07	103.43	7	MC	F	2						
4	Fall	13000	310	121019_08	97.5	8	PC	B	2						
4	Fall	13000	311	121019_09	40.47	9	PC	B	1						
4	Fall	13000	312	121019_10	37.11	10	PC	B	2						
4	Fall	13000	313	121019_11	35.37	11	PC	F	1						
4	Fall	13000	314	121019_12	43.37	12	PC	F	1						
4	Fall	13000	315	121019_13	105.12	13	PC	F	0						
4	Fall	13000	316	121019_14	104.37	14	PC	F	1						
4	Fall	13000	317	121019_15	40.64	15	PC	F	2						
4	Fall	13000	318	121019_16	43.78	16	PC	G	2						
4	Fall	13000	319	121103_01	35.35	1	SU	G	1						
4	Fall	13000	320	121103_02	37.95	2	SU	F	1						
4	Fall	13000	321	121103_03	106.41	3	MS	F	1						
4	Fall	13000	322	121103_04	107.6	4	SU	G	1						
4	Fall	13000	323	121103_05	41.87	5	SU	F	1						
4	Fall	13000	324	121103_06	42.34	6	PC	G	1						
4	Fall	13000	325	121103_07	103.08	7	MS	F	1						
4	Fall	13000	326	121103_08	99.4	8	PC	F	1						
4	Fall	13000	327	121103_09	40.51	9	PC	G	1						
4	Fall	13000	328	121103_10	36.27	10	MS	F	1						
4	Fall	13000	329	121103_11	36.31	11	PC	F	1						
4	Fall	13000	330	121103_12	44.15	12	MS	F	1						
4	Fall	13000	331	121103_13	105.18	13	PC	G	1						
4	Fall	13000	332	121103_14	104.09	14	SU	G	0	201	54.86	Cm	M	0	CB
4	Fall	13000	333	121103_15	43.4	16	PC	G	1	202	105.7	Cm	M	0	CB
4	Fall	13000	334	121103_16	39.55	15	PC	F	1	203	161.25	Cc	M	0	CB
4	Fall	13000	334	121103_16	39.55	15	PC	F	1	204	9.33	Cc	M	0	CB
4	Fall	13000	334	121103_16	39.55	15	PC	F	1	205	105.7	Cc	M	0	CB
4	Fall	13000	334	121103_16	39.55	15	PC	F	1	206	105.7	Cc	M	0	CB
4	Fall	13000	334	121103_16	39.55	15	PC	F	1	207	105.7	Cc	M	0	CB

Transect Dataset

Stratum	Line transect ID	Seson Year	Length	Transect Number	Cloud_Cover	Glare	Transect sea state
Winter	110128_01	Winter2011	84.2	1	MC	G	3
Winter	110128_02	Winter2011	92.6	2	MC	F	3
Winter	110129_03	Winter2011	53.71	3	SU	F	3
Winter	110129_04	Winter2011	52.5	4	SU	F	3
Winter	110129_05	Winter2011	84.26	5	SU	F	2
Winter	110129_06	Winter2011	54.21	6	MS	F	2
Winter	110129_07	Winter2011	50	7	MS	F	2
Winter	110129_08	Winter2011	50	8	MS	F	2
Winter	110129_09	Winter2011	79.64	9	MS	F	2
Winter	110129_10	Winter2011	79.64	10	MS	F	2
Winter	110129_11	Winter2011	50	11	MS	F	2
Winter	110129_12	Winter2011	50	12	PC	F	2
Winter	110226_01	Winter2011	91.2	1	PC	F	
Winter	110226_02	Winter2011	92.6	2	PC	F	
Winter	110226_03	Winter2011	52.49	3	MS	F	
Winter	110226_04	Winter2011	54.35	4	MS	F	
Winter	110226_05	Winter2011	86.67	5	MS	F	
Winter	110226_06	Winter2011	83.69	6	SU	F	
Winter	110226_07	Winter2011	50	7	MS	F	
Winter	110226_08	Winter2011	50	8	MS	F	
Winter	110226_09	Winter2011	83.42	9	MS	F	
Winter	110226_10	Winter2011	79.64	10	MS	F	
Winter	110226_11	Winter2011	50	11	MS	F	
Winter	110226_12	Winter2011	50	12	MS	F	
Winter	111216_01	Winter2011	96.17	1	PC	F	
Winter	111216_02	Winter2011	104.03	2	PC	F	
Winter	111216_03	Winter2011	56	3	PC	F	
Winter	111216_04	Winter2011	56.35	4	PC	F	
Winter	111216_05	Winter2011	77.53	5	PC	F	
Winter	111216_06	Winter2011	77.66	6	PC	F	
Winter	111216_07	Winter2011	60.74	7	PC	F	
Winter	111216_08	Winter2011	52.37	8	PC	F	
Winter	111216_09	Winter2011	89.1	9	PC	F	
Winter	111216_10	Winter2011	95.06	10	PC	F	
Winter	111216_11	Winter2011	56.51	11	PC	F	

Winter	111216_12	Winter2011	54.78	12	PC	F	
Winter	120301_01	Winter2012	35.13	1	PC	F	
Winter	120301_02	Winter2012	35.99	2	PC	F	
Winter	120301_03	Winter2012	105.86	3	PC	F	
Winter	120301_04	Winter2012	107.85	4	PC	F	
Winter	120301_05	Winter2012	41.08	5	PC	F	
Winter	120301_06	Winter2012	41.15	6	PC	F	
Winter	120301_07	Winter2012	102.06	7	MS	F	
Winter	120301_08	Winter2012	102.63	8	PC	F	
Winter	120301_09	Winter2012	40.48	9	MS	F	
Winter	120301_10	Winter2012	32.8	10	PC	F	
Winter	120301_11	Winter2012	33.79	11	PC	F	
Winter	120301_12	Winter2012	45.69	12	MS	F	
Winter	120301_13	Winter2012	104.71	13	PC	F	
Winter	120301_14	Winter2012	103.8	14	PC	F	
Winter	120301_15	Winter2012	38.73	15	MS	F	
Winter	120301_16	Winter2012	42.63	16	MS	F	
Winter	121216_01	Winter2012	36.95	1	MS	F	
Winter	121216_02	Winter2012	38.2	2	MS	F	
Winter	121216_03	Winter2012	105.96	3	MS	F	
Winter	121216_04	Winter2012	108.33	4	PC	F	
Winter	121216_05	Winter2012	42.96	5	MC	F	
Winter	121216_06	Winter2012	42.61	6	PC	F	
Winter	121216_07	Winter2012	102.23	7	MC	F	
Winter	121216_08	Winter2012	99.08	8	MC	F	
Winter	121216_09	Winter2012	39.01	9	SU	F	
Winter	121216_10	Winter2012	35.09	10	MS	F	
Winter	121216_11	Winter2012	35.36	11	MS	F	
Winter	121216_12	Winter2012	43.3	12	MS	F	
Winter	121216_13	Winter2012	105.7	13	MS	F	
Winter	121216_14	Winter2012	104.98	14	MS	F	
Winter	121216_15	Winter2012	37.67	15	SU	F	
Winter	121216_16	Winter2012	43.93	16	MS	F	
Winter	130116_01	Winter2012	36.95	1	MS	F	
Winter	130116_02	Winter2012	38.56	2	MS	F	
Winter	130116_03	Winter2012	106.93	3	MS	F	
Winter	130116_04	Winter2012	107.41	4	MS	F	

Winter	130116_05	Winter2012	43.04	5	PC	G	1
Winter	130116_06	Winter2012	43.63	6	PC	G	2
Winter	130116_07	Winter2012	104.19	7	PC	G	1
Winter	130116_08	Winter2012	98.61	8	PC	G	2
Winter	130116_09	Winter2012	40.92	9	PC	G	2
Winter	130116_10	Winter2012	36.07	10	PC	G	2
Winter	130116_11	Winter2012	37.12	11	PC	G	1
Winter	130116_12	Winter2012	44.33	12	PC	G	1
Winter	130116_13	Winter2012	105.82	13	PC	G	1
Winter	130116_14	Winter2012	104.89	14	PC	G	1
Winter	130116_15	Winter2012	41.39	15	PC	G	1
Winter	130116_16	Winter2012	44.88	16	PC	G	1
Spring	110325_01	Spring2011	90.16	1	PC	G	0
Spring	110325_02	Spring2011	102.79	2	PC	G	0
Spring	110325_03	Spring2011	55.65	3	PC	G	0
Spring	110325_04	Spring2011	58.21	4	PC	G	0
Spring	110325_05	Spring2011	78.65	5	PC	G	0
Spring	110325_06	Spring2011	76.59	6	PC	G	0
Spring	110325_07	Spring2011	59.66	7	PC	G	0
Spring	110325_08	Spring2011	53.75	8	PC	G	0
Spring	110325_09	Spring2011	90.12	9	PC	G	0
Spring	110325_10	Spring2011	96.01	10	PC	G	0
Spring	110325_11	Spring2011	56.18	11	MS	G	0
Spring	110325_12	Spring2011	57.81	12	PC	G	0
Spring	110507_01	Spring2011	86.49	1	PC	G	0
Spring	110507_02	Spring2011	86.49	2	PC	G	0
Spring	110507_03	Spring2011	57.95	3	PC	G	0
Spring	110507_04	Spring2011	56.84	4	PC	G	0
Spring	110507_05	Spring2011	76.85	5	MS	G	0
Spring	110507_06	Spring2011	76.12	6	MS	G	0
Spring	110507_07	Spring2011	59.23	7	SU	F	0
Spring	110507_08	Spring2011	55.05	8	MS	G	0
Spring	110507_09	Spring2011	92.33	9	SU	G	0
Spring	110507_10	Spring2011	98.61	10	PC	F	0
Spring	110507_11	Spring2011	57.43	11	SU	G	1
Spring	110507_12	Spring2011	55.33	12	PC	F	1
Spring	110527_01	Spring2011	97.96	1	PC	G	1

Spring	110527_02	Spring2011	101.91	2	PC	G	1
Spring	110527_03	Spring2011	57.27	3	PC	G	1
Spring	110527_04	Spring2011	57.63	4	PC	G	1
Spring	110527_05	Spring2011	78.2	5	PC	G	1
Spring	110527_06	Spring2011	78.15	6	PC	G	1
Spring	110527_07	Spring2011	59.86	7	MC	F	1
Spring	110527_08	Spring2011	55.05	8	MC	G	1
Spring	110527_09	Spring2011	80.56	9	MC	F	1
Spring	110527_10	Spring2011	84.27	10	MC	G	1
Spring	110527_11	Spring2011	57.22	11	MC	G	1
Spring	110527_12	Spring2011	55.86	12	MC	F	1
Spring	120324_01	Spring2012	35.68	1	PC	G	1
Spring	120324_02	Spring2012	37.54	2	PC	G	1
Spring	120324_03	Spring2012	107.14	3	MS	G	1
Spring	120324_04	Spring2012	109.2	4	PC	G	1
Spring	120324_05	Spring2012	41.59	5	PC	G	1
Spring	120324_06	Spring2012	43.34	6	PC	F	1
Spring	120324_07	Spring2012	102.56	7	MS	G	1
Spring	120324_08	Spring2012	104.46	8	MS	F	1
Spring	120324_09	Spring2012	41.88	9	PC	F	1
Spring	120324_10	Spring2012	33.19	10	MS	G	1
Spring	120324_11	Spring2012	33.91	11	MS	G	1
Spring	120324_12	Spring2012	34.22	12	MS	G	1
Spring	120324_13	Spring2012	103.53	13	MC	G	2
Spring	120324_14	Spring2012	101.74	14	MS	G	1
Spring	120324_15	Spring2012	38.71	15	PC	G	1
Spring	120324_16	Spring2012	41.9	16	PC	G	1
Spring	120505_01	Spring2012	35.67	1	MC	G	1
Spring	120505_02	Spring2012	38.58	2	PC	G	1
Spring	120505_03	Spring2012	106.56	3	PC	G	1
Spring	120505_04	Spring2012	108.65	4	PC	G	1
Spring	120505_05	Spring2012	42.4	5	PC	G	1
Spring	120505_06	Spring2012	44.02	6	PC	G	1
Spring	120505_07	Spring2012	103.24	7	PC	G	1
Spring	120505_08	Spring2012	105.41	8	PC	G	1
Spring	120505_09	Spring2012	40.58	9	PC	G	1
Spring	120505_10	Spring2012	34.04	10	PC	G	1

Spring	120505_11	Spring2012	35.09	11	PC	G	1
Spring	120505_12	Spring2012	43.5	12	PC	G	1
Spring	120505_13	Spring2012	105.03	13	PC	G	1
Spring	120505_14	Spring2012	104.54	14	PC	G	1
Spring	120505_15	Spring2012	39.73	15	PC	F	1
Spring	120505_16	Spring2012	43.95	16	PC	G	1
Spring	120519_03	Spring2012	71.14	3	MS	G	1
Spring	120519_04	Spring2012	107.68	4	PC	G	1
Spring	120519_05	Spring2012	79.77	5	MS	G	1
Spring	120519_06	Spring2012	82.74	6	PC	G	1
Spring	120519_07	Spring2012	60.45	7	PC	G	1
Spring	120519_08	Spring2012	63.08	8	SU	G	1
Spring	120519_09	Spring2012	40.38	9	SU	G	1
Spring	120519_10	Spring2012	36.02	10	SU	G	1
Spring	120519_11	Spring2012	35.33	11	SU	G	0
Spring	120519_12	Spring2012	42.85	12	MS	G	0
Spring	120519_13	Spring2012	105.66	13	SU	G	0
Spring	120519_14	Spring2012	105.05	14	SU	G	1
Spring	120519_15	Spring2012	41.95	15	MS	G	0
Spring	120519_16	Spring2012	44.09	16	MS	G	0
Summer	110617_01	Summer2011	96.03	1	MS	G	0
Summer	110617_02	Summer2011	103.4	2	PC	G	1
Summer	110617_03	Summer2011	57.12	3	MS	G	0
Summer	110617_04	Summer2011	58.61	4	PC	G	0
Summer	110617_05	Summer2011	75.85	5	PC	G	0
Summer	110617_06	Summer2011	78.55	6	PC	G	0
Summer	110617_07	Summer2011	58.71	7	MS	G	0
Summer	110617_08	Summer2011	56.53	8	MS	G	0
Summer	110617_09	Summer2011	90.14	9	MS	G	0
Summer	110617_10	Summer2011	96.38	10	PC	G	0
Summer	110617_11	Summer2011	29.89	11	PC	G	1
Summer	110617_12	Summer2011	54.78	12	PC	G	1
Summer	110715_01	Summer2011	86.49	1	PC	G	1
Summer	110715_02	Summer2011	103.4	2	PC	G	1
Summer	110715_03	Summer2011	57.12	3	PC	F	1
Summer	110715_04	Summer2011	58.61	4	PC	G	1
Summer	110715_05	Summer2011	75.85	5	PC	G	1

Summer	110715_06	Summer2011	78.55	6	PC	G	1
Summer	110715_07	Summer2011	58.71	7	PC	G	1
Summer	110715_08	Summer2011	56.53	8	PC	G	1
Summer	110715_09	Summer2011	89.73	9	PC	G	1
Summer	110715_10	Summer2011	97.46	10	PC	G	1
Summer	110715_11	Summer2011	49.82	11	SU	G	1
Summer	110715_12	Summer2011	54.78	12	SU	G	1
Summer	110813_01	Summer2011	97.71	1	MS	G	1
Summer	110813_02	Summer2011	103.22	2	MS		1
Summer	110813_03	Summer2011	56.52	3	MC	F	1
Summer	110813_04	Summer2011	57.93	4	MC	G	2
Summer	110813_05	Summer2011	77.78	5	MC	F	1
Summer	110813_06	Summer2011	78.33	6	MC	G	1
Summer	110813_07	Summer2011	59.58	7	MC	F	1
Summer	110813_08	Summer2011	53.06	8	PC	F	1
Summer	110813_09	Summer2011	90.05	9	PC	G	1
Summer	110813_10	Summer2011	97.26	10	PC	G	0
Summer	110813_11	Summer2011	56.89	11	PC	F	0
Summer	110813_12	Summer2011	57.07	12	PC	F	0
Summer	120701_01	Summer2012	33.68	1	PC	G	0
Summer	120701_02	Summer2012	38.72	2	PC	F	0
Summer	120701_03	Summer2012	106.09	3	PC	G	0
Summer	120701_04	Summer2012	107.56	4	PC	G	0
Summer	120701_05	Summer2012	42.62	5	SU	F	1
Summer	120701_06	Summer2012	44.56	6	SU	G	1
Summer	120701_07	Summer2012	102.86	7	SU	F	0
Summer	120701_08	Summer2012	102.79	8	SU	G	0
Summer	120701_09	Summer2012	31.97	9	SU	G	0
Summer	120701_10	Summer2012	34.38	10	SU	G	1
Summer	120701_11	Summer2012	36.19	11	SU	G	0
Summer	120701_12	Summer2012	43.93	12	MS	G	1
Summer	120701_13	Summer2012	105.29	13	MS	F	0
Summer	120701_14	Summer2012	102.51	14	MS	G	0
Summer	120701_15	Summer2012	40.67	15	MS	G	1
Summer	120701_16	Summer2012	41.47	16	MS	G	0
Summer	120728_01	Summer2012	31.47	1	MS	F	1
Summer	120728_02	Summer2012	37.72	2	MS	G	0

Summer	120728_03	Summer2012	106.55	3	MS	G	1
Summer	120728_04	Summer2012	108.42	4	MS	F	0
Summer	120728_05	Summer2012	42.23	5	PC	G	0
Summer	120728_06	Summer2012	42.62	6	PC	G	0
Summer	120728_07	Summer2012	102.52	7	SU	F	0
Summer	120728_08	Summer2012	98.31	8	SU	G	0
Summer	120728_09	Summer2012	39.93	9	PC	G	0
Summer	120728_10	Summer2012	33.11	10	PC	G	0
Summer	120728_11	Summer2012	36.29	11	PC	F	0
Summer	120728_12	Summer2012	42.82	12	PC	G	0
Summer	120728_13	Summer2012	104.68	13	PC	G	0
Summer	120728_14	Summer2012	104.91	14	PC	G	0
Summer	120728_15	Summer2012	40.4	15	PC	G	1
Summer	120728_16	Summer2012	42.08	16	PC	G	0
Summer	120811_01	Summer2012	34.01	1	PC	G	0
Summer	120811_02	Summer2012	36.83	2	SU	G	0
Summer	120811_03	Summer2012	105.09	3	MS	G	0
Summer	120811_04	Summer2012	107.04	4	SU	G	0
Summer	120811_05	Summer2012	40.31	5	MC	F	0
Summer	120811_06	Summer2012	41.16	6	MC	G	1
Summer	120811_07	Summer2012	101.97	7	MC	F	0
Summer	120811_08	Summer2012	98.52	8	MC	G	1
Summer	120811_09	Summer2012	40.84	9	PC	F	0
Summer	120811_10	Summer2012	35.19	10	MC	G	0
Summer	120811_11	Summer2012	35.73	11	PC	G	1
Summer	120811_12	Summer2012	41.82	12	MC	G	0
Summer	120811_13	Summer2012	104.11	13	MC	G	1
Summer	120811_14	Summer2012	103.47	14	MC	G	0
Summer	120811_15	Summer2012	38.82	15	MC	G	0
Summer	120811_16	Summer2012	41.99	16	PC	G	0
Fall	110916_01	Fall2011	98.12	1	MC	G	1
Fall	110916_02	Fall2011	103.78	2	PC	F	0
Fall	110916_03	Fall2011	56.4	3	MC	G	0
Fall	110916_04	Fall2011	58.06	4	PC	F	0
Fall	110916_05	Fall2011	76.86	5	PC	F	2
Fall	110916_06	Fall2011	77.18	6	PC	F	2
Fall	110916_07	Fall2011	59.52	7	PC	G	1

Fall	110916_08	Fall2011	52.86	8	PC	G	1
Fall	110916_09	Fall2011	89.14	9	PC	G	1
Fall	110916_10	Fall2011	97.04	10	MS	G	1
Fall	110916_11	Fall2011	57.31	11	MS	G	0
Fall	110916_12	Fall2011	57.45	12	SU	G	1
Fall	111014_01	Fall2011	96.64	1	MS	F	0
Fall	111014_02	Fall2011	105.29	2	PC	F	1
Fall	111014_03	Fall2011	55.82	3	PC	B	1
Fall	111014_04	Fall2011	58.12	4	PC	F	1
Fall	111014_05	Fall2011	78.18	5	MS	G	0
Fall	111014_06	Fall2011	79.86	6	SU	F	0
Fall	111014_07	Fall2011	60.26	7	PC	G	1
Fall	111014_08	Fall2011	54.13	8	MS	F	1
Fall	111014_09	Fall2011	49.2	9	MS	G	0
Fall	111014_10	Fall2011	96.72	10	PC	F	1
Fall	111014_11	Fall2011	56.84	11	PC	G	0
Fall	111014_12	Fall2011	56.88	12	MC	B	1
Fall	111123_01	Fall2011	98.89	1	PC	G	0
Fall	111123_02	Fall2011	103.73	2	PC	G	1
Fall	111123_03	Fall2011	54.18	3	PC	G	0
Fall	111123_04	Fall2011	57.98	4	PC	F	0
Fall	111123_05	Fall2011	78.1	5	PC	F	0
Fall	111123_06	Fall2011	78.88	6	PC	G	0
Fall	111123_07	Fall2011	60.07	7	PC	F	1
Fall	111123_08	Fall2011	52.56	8	PC	G	1
Fall	111123_09	Fall2011	86.62	9	PC	F	0
Fall	111123_10	Fall2011	96.56	10	PC	G	0
Fall	111123_11	Fall2011	56.66	11	PC	F	0
Fall	111123_12	Fall2011	56.61	12	PC	G	1
Fall	120901_01	Fall2012	34.5	1	SU	G	2
Fall	120901_02	Fall2012	39.71	2	SU	F	2
Fall	120901_03	Fall2012	105.98	3	SU	F	0
Fall	120901_04	Fall2012	107.26	4	SU	G	1
Fall	120901_05	Fall2012	41.6	5	SU	G	0
Fall	120901_06	Fall2012	43.02	6	SU	G	1
Fall	120901_07	Fall2012	103.31	7	SU	G	0
Fall	120901_08	Fall2012	104.5	8	SU	F	1

Fall	120901_09	Fall2012	39.41	9	SU	F	0
Fall	120901_10	Fall2012	35.81	10	SU	F	2
Fall	120901_11	Fall2012	36.15	11	SU	F	0
Fall	120901_12	Fall2012	42.77	12	SU	F	2
Fall	120901_13	Fall2012	104.41	13	SU	F	0
Fall	120901_14	Fall2012	103.61	14	SU	F	0
Fall	120901_15	Fall2012	39.52	15	SU	G	0
Fall	120901_16	Fall2012	42.5	16	SU	G	1
Fall	121019_01	Fall2012	34.54	1	MC	G	2
Fall	121019_02	Fall2012	37.35	2	PC	G	2
Fall	121019_03	Fall2012	106.77	3	MC	G	3
Fall	121019_04	Fall2012	105.57	4	PC	G	2
Fall	121019_05	Fall2012	41.87	5	MC	G	3
Fall	121019_06	Fall2012	43.46	6	PC	G	2
Fall	121019_07	Fall2012	103.43	7	MC	F	2
Fall	121019_08	Fall2012	97.5	8	PC	B	2
Fall	121019_09	Fall2012	40.47	9	PC	B	1
Fall	121019_10	Fall2012	37.11	10	PC	B	2
Fall	121019_11	Fall2012	35.37	11	PC	F	1
Fall	121019_12	Fall2012	43.37	12	PC	F	1
Fall	121019_13	Fall2012	105.12	13	PC	F	0
Fall	121019_14	Fall2012	104.37	14	PC	F	1
Fall	121019_15	Fall2012	40.64	15	PC	F	2
Fall	121019_16	Fall2012	43.78	16	PC	G	2
Fall	121103_01	Fall2012	35.35	1	SU	G	1
Fall	121103_02	Fall2012	37.95	2	SU	F	1
Fall	121103_03	Fall2012	106.41	3	MS	F	1
Fall	121103_04	Fall2012	107.6	4	SU	G	1
Fall	121103_05	Fall2012	41.87	5	SU	F	1
Fall	121103_06	Fall2012	42.34	6	PC	G	1
Fall	121103_07	Fall2012	103.08	7	MS	F	1
Fall	121103_08	Fall2012	99.4	8	PC	F	1
Fall	121103_09	Fall2012	40.51	9	PC	G	1
Fall	121103_10	Fall2012	36.27	10	MS	F	1
Fall	121103_11	Fall2012	36.31	11	PC	F	1
Fall	121103_12	Fall2012	44.15	12	MS	F	1
Fall	121103_13	Fall2012	105.18	13	PC	G	1

Fall	121103_14	Fall2012	104.09	14	SU	G	0
Fall	121103_15	Fall2012	43.4	16	PC	G	1
Fall	121103_16	Fall2012	39.55	15	PC	F	1

Observations Dataset

Line transect ID	Perp distance	Obs Index No	Species	Size	Latitude	Longitude	Sea State	Observer	Behavior	Direction
110226_02	259.4	7	U	U	26.57483	-80.0073	0	EM		
111216_03	86.81	143	Cc	M	26.50005	-80.0342	1	SEC	S	S
111216_03	86.81	143	Cc	M	26.50005	-80.0342	1	SEC	S	S
120301_04	50.13	149	U	S	26.51777	-79.4017	1	CB	In weed line	
120301_06	75.4	152	Cc	S	26.38495	-79.7285	1	CB		
120301_11	54.86	153	Cc	M	26.04881	-79.8564	1	JP	S	S
120301_12	36.76	154	Cc	M	25.98429	-80.0964	1	CB	S	S
120301_12	16.71	155	Cc	S	25.98349	-80.1079	1	CB	S	S
120301_13	12.98	156	Cc	L	25.92093	-80.0345	1	CB	S	N
120301_13	36.76	157	Cm	S	25.92084	-79.2064	1	CB	S	NE
120301_14	112.55	160	U	S	25.85162	-79.671	1	CB	Floating	
121216_01	70	386	Cm	S	25.71673	-80.0978	2	CB	S	SE
121216_07	161.25	384	Cm	M	26.31654	-79.9467	2	CB	S	E
121216_11	75.4	385	Cm	L	26.04935	-79.8781	2	CB	S	S
130116_16	112.55	390	Cm	S	25.71916	-80.1334	0	CB	S	NE
110507_03	99.15	11	Cc	U	26.49916	-79.9874	1	EM		
110507_04	70	14	Cc	M	26.41548	-80.0571	2	EM	B	
110507_05	259.4	15	U	M	26.3299	-80.0476	1	SEC	S	N
110507_05	335.62	16	Cc	U	26.33437	-80.0205	2	EM	S	SE
110507_05	203.87	17	U	M	26.33437	-80.0205	2	SEC	S	W
110507_06	313.97	18	U	S	26.26812	-80.0216	2	EM	U	N
110507_06	70	19	U	M	26.26887	-80.0426	2	SEC	S	N
110507_06	99.15	20	U	M	26.26887	-80.0426	2	SEC	S	N
110507_06	170.97	21	U	M	26.26805	-80.0505	2	SEC	D	
110507_07	135.08	22	U	M-L	26.18462	-80.0404	2	SEC	S	S
110507_07	181.27	23	Cc	M-L	26.18462	-80.0404	2	SEC	S	S
110507_07	99.15	24	U	M-L	26.18798	-80.0196	2	SEC	S	S
110507_07	294.21	25	U	U	26.18691	-79.9384	2	EM	S	SW
110507_07	99.15	26	U	L	26.1867	-79.8418	2	SEC	D	
110507_08	70	28	Cc	L	26.12413	-79.9039	2	SEC	S	N
110507_08	243.97	30	U	U	26.12502	-79.9162	2	EM	S	
110507_08	135.08	31	Cc	M	26.11846	-79.9837	2	SEC	S	S
110507_08	70	32	Cc	M-L	26.11384	-80.0166	2	SEC	S	N
110507_10	335.62	36	U	M	25.9667	-79.9924	2	SEC		
110507_11	112.55	38	U	U	25.8854	-80.0097	1	SEC	U	
110507_11	70	39	Cc	M	25.8854	-80.0097	1	SEC	D	S
110507_11	243.97	40	U	M	25.88477	-79.9838	1	SEC	S	S
110527_01	294.21	42	Cm	M	26.65319	-79.931	1	JP	S	W

110527_04	127.21	46	Cm	M-L	26.41664	-79.8348	1	JP	S	E
110527_04	216.32	47	Cm	L			1	JP	S	S
110527_06	143.35	50	U	M-L	26.26634	-79.9659	1	JP	S	E
110527_07	119.71	51	Cc	L	26.18565	-80.0737	2	JP	S	S
110527_07	5.76	52	Cm	M-L	26.18228	-79.866	2	JP	S	S
110527_12	135.08	54	Cm	S-M	25.80308	-80.0856	2	JP	U	E
110527_12	92.86	56	Cc	M-L	25.80117	-79.7447	2	JP	U	E
120324_02	86.81	163	Cm	S	26.65019	-79.9941	0	CB	S	E
120324_10	161.25	168	U	M	26.1147	-79.9993	0	CB	S	S
120324_13	86.81	169	Cm	M	25.91946	-80.0554	0	SEC	S	W
120324_13	143.35	170	Cc	M	25.9171	-79.8174	0	SEC	S	W
120324_13	54.86	171	Cc	L	25.9168	-79.3593	0	CB	S	W
120324_14	192.22	173	Cc	S	25.85092	-79.5722	0	SEC	S	S
120324_14	54.86	174	Cc	M	25.84891	-80.0565	1	SEC	S	SE
120324_15	50.13	175	Cm	M	25.78492	-80.0804	0	CB	S	W
120324_15	229.65	177	Cc	S	25.78619	-79.9955	0	SEC	S	W
120505_02	99.15	179	Lk	S	26.65069	-79.7634	1	CB	S	E
120505_03	170.97	180	Cm	M	26.58461	-79.9775	1	CB	S	S
120505_04	99.15	182	Cc	L	26.51713	-79.1675	1	SEC	S	NE
120505_04	9.33	184	Cc	L	26.51773	-79.8944	1	CB	S	E
120505_04	112.55	185	Cc	M	26.51667	-79.9586	1	CB	S	N
120505_05	119.71	186	U	S-M	26.45174	-80.0502	1	SEC	S	N
120505_05	119.71	187	U	S-M	26.45192	-80.0459	1	SEC	S	NW
120505_05	135.08	188	U	M	26.45197	-80.0418	1	SEC	S	S
120505_05	59.74	189	Cc	L	26.45229	-79.9859	1	CB	S	S
120505_05	75.4	190	Cc	L	26.453	-79.9424	1	CB	Swimming close	S
120505_05	75.4	190	Cc	L	26.453	-79.9424	1	CB	Swimming close	S
120505_05	135.08	191	Cc	M	26.45215	-79.9226	1	SEC	S	S
120505_06	70	193	Cc	L	26.38422	-79.995	1	SEC		NW
120505_06	119.71	194	Cc	M	26.38418	-80.0346	1	CB	S	E
120505_07	28.43	195	Cc	M	26.318	-80.0529	1	CB	S	S
120505_07	112.55	196	Cc	L	26.31684	-80.0169	1	SEC	S	SE
120505_07	243.97	197	Cc	L	26.31722	-79.925	1	SEC	S	ESE
120505_08	112.55	199	Cc	L	26.25041	-79.4166	1	SEC		S
120505_09	45.54	201	Cc	L	26.18326	-80.0209	1	SEC		E
120505_09	99.15	202	Cc	L	26.18365	-80.0036	1	SEC	S	S
120505_12	216.32	204	Cc	M	25.982	-79.9425	1	CB	S	S
120505_13	24.43	205	Cc	M	25.91772	-80.0809	1	CB	S	S
120505_13	24.43	206	Cc	L	25.91662	-80.0588	1	SEC	S	W
120505_13	161.25	207	Cc	S	25.91667	-79.8015	1	CB	S	W

120505_14	119.71	209	Cc	M	25.85043	-80.05	1	CB	S	E
120505_15	216.32	210	Cc	M	25.78389	-80.0563	1	CB	S	N
120505_15	243.97	211	U	M	25.78379	-80.052	1	SEC		SW
120505_16	119.71	214	Cc	M	25.71808	-79.9969	1	CB	S	E
120505_16	181.27	215	U		25.71843	-80.031	1	SEC	S	S
120505_16	135.08	216	Cc		25.71741	-80.09	1	CB	Mating	
120505_16	135.08	216	Cc		25.71741	-80.09	1	CB	Mating	
120505_16	45.54	217	Cc		25.71794	-80.1073	1	SEC	S	SW
120519_04	41.09	219	Cc	M	26.51525	-79.8178	2	CB	Diving	
120519_04	50.13	220	Cc	S	26.51643	-79.7939	1	SEC	S	W
120519_05	45.54	221	Cc	M	26.38263	-79.9649	0	SEC	S	W
120519_05	12.98	222	Cc	M	26.38362	-79.8882	0	SEC	Diving	
120519_07	12.98	223	Cc	M	26.32326	-79.912	1	SEC	S	S
120519_07	75.4	224	Cc	S	26.31746	-79.9767	0	SEC	S	E
120519_08	119.71	225	Cm	M	26.24805	-80.0243	1	CB	S	S
120519_09	203.87	226	Dc	L	26.18551	-80.0141	0	CB	S	S
120519_09	161.25	227	Cc	M	26.18457	-80.0605	0	CB	S	SE
120519_09	192.22	228	Cc	M	26.18391	-80.0726	0	SEC		
120519_09	41.09	229	Cc	L	26.18387	-80.0748	0	CB	S	E
120519_10	12.98	230	Cc	L	26.1198	-80.0091	0	CB	S	N
120519_10	127.21	231	Cc	S	26.12275	-79.768	0	SEC		
120519_11	70	233	Cc	M	26.05225	-79.8791	0	CB	S	NE
120519_12	28.43	235	Cc	S	25.99113	-80.1079	0	CB	S	S
120519_13	75.4	238	Cc	S	25.91871	-79.2703	0	SEC		
120519_13	119.71	243	Cc	S	25.91688	-79.9521	0	SEC		
120519_13	41.09	244	Cc	M	25.91668	-80.0618	0	SEC		
120519_13	20.53	245	Cm	M	25.91629	-80.0822	0	SEC		
120519_13	36.76	246	Cm	L	25.91675	-80.0933	0	SEC		
120519_13	20.53	247	Cc	S	25.91764	-80.1082	0	CB	S	E
120519_14	12.98	248	Cc	S	25.84951	-80.1024	0	CB	S	W
120519_14	32.54	249	Cc	S	25.84692	-79.9976	0	SEC		
120519_14	54.86	250	Cc	S	25.85279	-79.8216	0	SEC		
120519_14	64.78	251	Cc	S	25.85216	-79.6702	0	SEC		
120519_14	181.27	252	Cc	L	25.85257	-79.3482	0	SEC		
120519_15	161.25	257	Cm	M	25.78523	-80.0641	0	SEC		
120519_15	54.86	258	Cc		25.78463	-80.1043	0	SEC		
120519_16	75.4	260	Cm	M	25.7149	-80.1267	0	CB	S	S
120519_16	59.74	262	Cc	M	25.71582	-80.1128	0	CB	S	S
120519_16	86.81	263	Cc	M	25.71582	-80.1128	0	SEC		
120519_16	54.86	264	Cc	M	25.71598	-80.1093	0	CB	S	S

110617_02	161.25	59	Cm	M-L	26.57156	-80.0032	1	JP	S	E
110617_02	99.15	60	U	S	26.57314	-80.0218	1	JP		
110617_02	276.09	61	U	U	26.57328	-80.0251	1	JP		
110617_03	143.35	63	Cc	U	26.497	-80.034	1	JP		
110617_03	135.08	64	Cc	L	26.497	-80.034	1	JP	S	W
110617_03	181.27	66	Cm	M-L	26.50332	-79.9143	1	JP		
110617_03	64.78	67	U	S	26.50254	-79.7748	1	JP	D	
110617_04	45.54	68	Cc	L	26.41508	-80.0317	1	JP		
110617_04	152.06	69	U	M	26.41601	-80.0454	1	JP	S	
110617_04	143.35	70	U	M	26.41601	-80.0454	1	JP	S	
110617_04	135.08	71	U	U	26.41642	-80.0533	1	EM	D	
110617_05	5.76	72	Cm	S-M	26.33641	-80.043	1	JP	S	W
110617_05	64.78	73	Cm	L	26.33394	-80.0038	1	JP	S	S
110617_06	294.21	75	U	S-M	26.26882	-79.8748	1	JP	S	S
110617_06	170.97	76	Cm	M-L	26.26721	-80.0069	1	JP	S	E
110617_07	32.54	77	Cm	M	26.18124	-80.0688	1	JP	S	S
110617_07	152.06	78	Cm	M	26.1864	-79.9441	1	JP		
110617_10	203.87	81	Cm	L	25.96678	-79.4776	1	JP	D	
110715_02	119.71	84	Cc	L	26.56489	-79.5047	1	JP	D	N
110715_02	50.13	85	Cc	M	26.56841	-79.5972	2	SEC	S	
110715_03	86.81	86	Cc	L	26.50044	-80.0368	1	SEC	S	S
110715_03	105.7	87	Cc	M	26.50049	-80.0081	1	SEC	S	S
110715_03	99.15	88	Cm	n/a	26.50199	-79.6702	1	JP	D	S
110715_04	75.4	89	Cm	S	26.41848	-80.0566	1	JP	S	N
110715_05	45.54	90	Cm	M	26.32883	-80.0624	1	SEC	S	
110715_06	119.71	92	Cm	M	26.26622	-79.8595	1	JP	Basking	E
110715_12	70	97	U	M	25.80509	-80.1096	1	SEC	S	
110813_03	143.35	99	Cm	M	26.50157	-79.785	0	JP	S	S
110813_05	64.78	102	U	M	26.33543	-80.0174	1	SEC	S	E
110813_08	81	107	Cc	L	26.12146	-79.8734	1	SEC	S	W
120701_03	359.44	269	Dc	L	26.58477	-79.6685	0	CB	S	S
120701_04	64.78	273	Cm	M	26.52046	-80.0301	0	CB	S	S
120701_05	243.97	275	U	S	26.45063	-80.0169	0	CB	S	S
120701_06	259.4	278	Cm	M	26.38713	-80.056	1	SEC	S	
120701_07	203.87	279	Cc	M	26.31521	-80.0125	0	SEC	S	NE
120701_08	16.71	280	Cm	L	26.24841	-79.596	0	CB	S	W
120701_09	135.08	283	Cm	M	26.1821	-80.0667	0	CB	S	S
120701_09	294.21	284	Cm	M	26.18301	-80.0818	0	SEC	S	W
120701_14	70	298	Cm	M/L	25.8508	-80.0875	1	SEC	S	NE
120701_15	32.54	299	Cm	M	25.78596	-80.0958	1	CB	S	S

120701_16	75.4	303	U	L	25.72899	-80.0661	0	CB	S	E
120728_01	36.76	305	Cm	M	26.7165	-79.8259	0	CB	S	S
120728_03	81	309	Cm	M	26.59568	-80.0335	0	SEC	S	E
120728_03	70	310	Cc	L	26.58525	-79.9671	0	CB	S	S
120728_05	20.53	316	U	S	26.45208	-80.0469	0	CB	S	S
120728_05	75.4	317	Cm	M	26.45127	-80.0421	0	CB	S	S
120728_06	64.78	319	Cc	S	26.3836	-79.9576	0	SEC	S	E
120728_06	12.98	320	Cc	L	26.38441	-80.0473	0	CB	S	S
120728_09	229.65	324	Cc	M	26.18805	-80.0743	0	CB	S	S
120728_09	112.55	325	Cc	M	26.18721	-80.065	0	CB	S	S
120728_09	192.22	326	Cm	M	26.18595	-80.0563	0	SEC	S	E
120728_09	203.87	327	U	M	26.18425	-79.996	0	SEC	S	N
120728_11	192.22	331	Cm	M	26.05328	-79.7859	1	SEC	S	S
120728_13	119.71	334	Cm	M	25.91664	-79.2397	0	SEC	S	S
120728_14	75.4	336	U	S	25.85025	-79.4158	0	SEC	S	N
120728_15	99.15	337	Cc	M	25.78338	-80.1047	0	CB	S	S
120728_15	81	338	Cm	M	25.78235	-80.0868	0	CB	S	S
120811_02	2.25	341	Cm	L	26.6513	-79.7983	0	CB	S	W
120811_02	64.78	342	Cc	S	26.6508	-79.8112	1	SEC	S	E
120811_03	143.35	343	Cm	M	26.58421	-79.9723	0	CB	S	S
120811_03	143.35	344	Cc	S	26.58333	-79.1588	1	SEC	S	SW
120811_04	59.74	345	Cc	M	26.51676	-80.0276	1	CB	Floating	
120811_06	105.7	346	Cm	S	26.3835	-79.8076	0	CB	S	SW
120811_06	32.54	347	Cc	M	26.38237	-80.053	0	CB	S	NE
120811_07	86.81	348	Cc	M	26.31579	-80.0428	1	SEC	S	W
120811_07	70	349	Cc		26.31606	-79.4103	1	SEC	S	SW
120811_10	127.21	351	Cm	M	26.11728	-79.9972	0	CB	S	NE
110916_02	75.4	117	CM	M	26.56967	-79.3156	0	SEC	S	E
110916_05	203.87	121	U	S	26.33293	-79.9882	0	SEC	S	W
110916_05	75.4	122	Cm	L	26.3336	-79.9745	0	JP	S	W
110916_10	203.87	128	U	S	25.96563	-79.6935	0	SEC	S	N
110916_10	181.27	129	Dc		25.96634	-79.7522	0	SEC	S	S
110916_11	24.43	131	U	S	25.88199	-80.0916	0	SEC	S	S
110916_11	112.55	132	U	M	25.88135	-79.9317	0	SEC	S	N
111014_07	135.08	135	Cc	M	26.1808	-79.8861	1	SEC	U	W
111123_12	335.62	140	Cc	M	25.80512	-80.0494	1	SEC	S	SW
111123_12	181.27	141	Cc	M	25.80051	-80.1039	1	SEC	S	S
120901_03	86.81	353	Cc	S	26.5842	-79.6302	1	SEC	S	W
120901_03	32.54	355	Cc	L	26.58301	-79.4612	1	CB	S	SW
120901_07	152.06	356	Cm	M	26.31596	-80.0545	0	CB	S	SE

120901_07	70	357	Cm	L	26.31675	-79.7307	0	SEC	D	E
121019_03	32.54	360	Cc	M	26.58234	-79.1875	1	CB	S	W
121103_14	54.86	376	Cm	M	25.91637	-79.2534	0	CB	S	SW
121103_15	105.7	377	Cm	M	25.84902	-80.0633	0	CB	S	S
121103_16	161.25	380	Cc	M	25.72246	-80.1128	0	CB	S	E
121103_16	9.33	381	Cc	M	25.72318	-80.1175	0	CB	S	S
121103_16	105.7	382	Cc	M	25.72571	-80.1295	0	CB	Floating	
121103_16	105.7	382	Cc	M	25.72571	-80.1295	0	CB	Floating	
121103_16	105.7	382	Cc	M	25.72571	-80.1295	0	CB	Floating	

Key for Abbreviations

Coud Cover	MC	mostly cloudy = >75% cloud cover
	PC	partly cloudy = 25-74% cloud cover
	MS	mostly sunny = 25-15% cloud cover
	SU	sunny = <15% cloud cover
Glare	B=bad, F=fair, P=poor	
Observers	EM = Erin McMichael- Primary Observer	
	JP = Justin Perrault- Primary Observer	
	CB = Caitlin Bovery - Primary Observer	
	SEC = Secondary Observers - Grouped for analyses	
Species	Cc = loggerhead	
	Cm = green	
	Lk = Kemp's ridley	
	Ei = Hawksbill	
	Dc = leatherback	
	U = unknown	
Turtle Size	S = small	
	M = medium	
	L = large	
	U = unknown	
Behavoir	S = Swimming	
	D = Diving	
	U = Unknown	
